# A genome-wide association study with tissue transcriptomics identifies genetic drivers for classic bladder exstrophy

**DOI:** 10.1038/s42003-022-04092-3

**Published:** 2022-11-09

**Authors:** Enrico Mingardo, Glenda Beaman, Philip Grote, Agneta Nordenskjöld, William Newman, Adrian S. Woolf, Markus Eckstein, Alina C. Hilger, Gabriel C. Dworschak, Wolfgang Rösch, Anne-Karolin Ebert, Raimund Stein, Alfredo Brusco, Massimo Di Grazia, Ali Tamer, Federico M. Torres, Jose L. Hernandez, Philipp Erben, Carlo Maj, Jose M. Olmos, Jose A. Riancho, Carmen Valero, Isabel C. Hostettler, Henry Houlden, David J. Werring, Johannes Schumacher, Jan Gehlen, Ann-Sophie Giel, Benedikt C. Buerfent, Samara Arkani, Elisabeth Åkesson, Emilia Rotstein, Michael Ludwig, Gundela Holmdahl, Elisa Giorgio, Alfredo Berettini, David Keene, Raimondo M. Cervellione, Nina Younsi, Melissa Ortlieb, Josef Oswald, Bernhard Haid, Martin Promm, Claudia Neissner, Karin Hirsch, Maximilian Stehr, Frank-Mattias Schäfer, Eberhard Schmiedeke, Thomas M. Boemers, Iris A. L. M. van Rooij, Wouter F. J. Feitz, Carlo L. M. Marcelis, Martin Lacher, Jana Nelson, Benno Ure, Caroline Fortmann, Daniel P. Gale, Melanie M. Y. Chan, Kerstin U. Ludwig, Markus M. Nöthen, Stefanie Heilmann, Nadine Zwink, Ekkehart Jenetzky, Benjamin Odermatt, Michael Knapp, Heiko Reutter

**Affiliations:** 1 Institute for Anatomy and Cell Biology, University Hospital Bonn, University of Bonn, Bonn, Germany; 2 Institute for Neuroanatomy, University Hospital Bonn, University of Bonn, Bonn, Germany; 3 Institute of Human Genetics, School of Medicine & University Hospital Bonn, University of Bonn, Bonn, Germany; 4 Centre for Genomic Medicine, University of Manchester, Manchester, UK; 5 Institute of Cardiovascular Regeneration, Centre for Molecular Medicine, Goethe University, Frankfurt am Main, Germany; 6 Georg-Speyer-Haus, Frankfurt am Main, Germany; 7 Department of Women´s and Children´s Health and Center for Molecular Medicine, Karolinska Institutet, Stockholm, Sweden; 8 Pediatric Surgery, Astrid Lindgren Children Hospital, Karolinska University Hospital, Stockholm, Sweden; 9 Division of Cell Matrix Biology and Regenerative Medicine, Faculty of Biology Medicine and Health, School of Biological Sciences, University of Manchester, Manchester, UK; 10 Royal Manchester Children’s Hospital, Manchester University NHS Foundation Trust, Manchester Academic Health Science Centre, Manchester, UK; 11 Institute of Pathology, University Hospital Erlangen, Friedrich-Alexander-University Erlangen-Nürnberg, Erlangen, Germany; 12 Comprehensive Cancer Center Erlangen-EMN (CCC ER-EMN), Erlangen, Germany; 13 BRIDGE-Consortium Germany e.V., Mannheim, Germany; 14 Department of Pediatrics and Adolescent Medicine, University Hospital Erlangen, Erlangen, Germany; 15 Department of Pediatric Urology, Clinic St. Hedwig, University Medical Center of Regensburg, Regensburg, Germany; 16 Department of Urology and Pediatric Urology, University Hospital of Ulm, Ulm, Germany; 17 Center for Pediatric, Adolescent and Reconstructive Urology, University Medical Center Mannheim, Heidelberg University, Mannheim, Germany; 18 Department of Medical Sciences and Medical Genetics Unit, Città della Salute e della Scienza University Hospital, University of Torino, Torino, Italy; 19 Institute for Maternal and Child Health, IRCCS Burlo Garofalo, Trieste, Italy; 20 Translational Pediatrics and Infectious Diseases, Hospital Clínico Universitario de Santiago, Santiago de Compostela, Spain; 21 Department of Internal Medicine, Hospital U M Valdecilla, University of Cantabria, IDIVAL, Santander, Spain; 22 Department of Urology and Urosurgery, Medical Faculty Mannheim, University of Heidelberg, Mannheim, Germany; 23 Institute of Genomic Statistics and Bioinformatics, University of Bonn, Bonn, Germany; 24 Department of Internal Medicine. Hospital U M Valdecilla, University of Cantabria, IDIVAL, Santander, Spain; 25 Stroke Research Centre, University College London, Institute of Neurology, London, UK; 26 Neurogenetics Laboratory, The National Hospital of Neurology and Neurosurgery, London, UK; 27 Department of Neurosurgery, Klinikum rechts der Isar, Technical University Munich, Munich, Germany; 28 Neurogenetics Laboratory, The National Hospital of Neurology and Neurosurgery, London, UK; 29 Stroke Research Center, Department of Brain Repair and Rehabilitation, UCL Institute of Neurology and The National Hospital for Neurology and Neurosurgery, London, UK; 30 Institute for Human Genetics, University of Marburg, Marburg, Germany; 31 Department of Women’s and Children’s Health and Center for Molecular Medicine, Bioclinicum, Karolinska Institutet, Stockholm, Sweden; 32 Department of Urology, Danderyds Hospital, Danderyd, Sweden; 33 Division of Neurogeriatrics, Department of Neurobiology, Care Sciences and Society, Karolinska Institutet, Stockholm, Sweden; 34 R&D Unit, Stockholms Sjukhem, Stockholm, Sweden; 35 ME Gynecology and Reproduction Medicine, Karolinska University Hospital, and Dept of Clintec, Karolinska Institutet, Stockholm, Sweden; 36 Department of Clinical Chemistry and Clinical Pharmacology, University of Bonn, Bonn, Germany; 37 Department of Pediatric Surgery, Queen Silvia Children’s Hospital, Gothenburg, Sweden; 38 Department of Molecular Medicine, University of Pavia, Pavia, Italy; 39 Laboratory of Molecular Medicine and Cytogenetics, IRCCS Mondino Foundation, Pavia, Italy; 40 Pediatric Urology Unit, Fondazione IRCCS Ca’ Granda Ospedale Maggiore Policlinico, Milan, Italy; 41 Paediatric Urology, Royal Manchester Children’s Hospital, Central Manchester University Hospitals NHS Foundation Trust, Manchester, UK; 42 Department for Pediatric Urology, Ordensklinikum Linz, Hospital of the Sisters of Charity, Linz, Austria; 43 Division of Pediatric Urology, Department of Urology, University of Erlangen-Nürnberg, Erlangen, Germany; 44 Department of Pediatric Surgery and Urology, Klinik Hallerwiese-Cnopfsche Kinderklinik, Nürnberg, Germany; 45 Department of Urology and Pediatric Urology, University Hospital Erlangen, Friedrich-Alexander-University Erlangen-Nürnberg, Erlangen, Germany; 46 Clinic for Paediatric Surgery and Paediatric Urology, Klinikum Bremen-Mitte, Bremen, Germany; 47 Department of Pediatric Surgery and Urology, University Hospital Cologne, Cologne, Germany; 48 Department of Pediatric Surgery, University of Leipzig, Leipzig, Germany; 49 Department for Health Evidence, Radboud Institute for Health Sciences, Radboud University Medical Center, Nijmegen, Netherlands; 50 Department of Urology, Pediatric Urology Center, Radboud University Nijmegen Medical Center, Nijmegen, The Netherlands; 51 Department of Genetics, Radboud University Nijmegen Medical Center, Nijmegen, The Netherlands; 52 Center of Pediatric Surgery Hannover, Hannover Medical School, Hannover, Germany; 53 Department of Renal Medicine, University College London, London, UK; 54 Department of Genomics, Life & Brain Center, University of Bonn, Bonn, Germany; 55 Department of Child and Adolescent Psychiatry, University Medical Center of the Johannes Gutenberg University Mainz, Mainz, Germany; 56 Faculty of Health, School of Medicine, University of Witten/Herdecke, Witten, Germany; 57 Institute of Medical Biometry, Informatics, and Epidemiology, University of Bonn, Bonn, Germany; 58 Division of Neonatology and Pediatric Intensive Care Medicine, Department of Pediatric and Adolescent Medicine, Friedrich-Alexander-University Erlangen-Nürnberg, Erlangen, Germany

**Keywords:** Genome-wide association studies, Gene expression

## Abstract

Classic bladder exstrophy represents the most severe end of all human congenital anomalies of the kidney and urinary tract and is associated with bladder cancer susceptibility. Previous genetic studies identified one locus to be involved in classic bladder exstrophy, but were limited to a restrict number of cohort. Here we show the largest classic bladder exstrophy genome-wide association analysis to date where we identify eight genome-wide significant loci, seven of which are novel. In these regions reside ten coding and four non-coding genes. Among the coding genes is EFNA1, strongly expressed in mouse embryonic genital tubercle, urethra, and primitive bladder. Re-sequence of EFNA1 in the investigated classic bladder exstrophy cohort of our study displays an enrichment of rare protein altering variants. We show that all coding genes are expressed and/or significantly regulated in both mouse and human embryonic developmental bladder stages. Furthermore, nine of the coding genes residing in the regions of genome-wide significance are differentially expressed in bladder cancers. Our data suggest genetic drivers for classic bladder exstrophy, as well as a possible role for these drivers to relevant bladder cancer susceptibility.

## Abstract (web summary)

A genome-wide association study on classic bladder exstrophy reveals eight genome-wide significant loci, most of which contained genes expressed in embryonic developmental bladder stages.

## Introduction

The bladder exstrophy-epispadias complex (BEEC) is a spectrum of congenital abnormalities which involves the abdominal wall, bony pelvis, the urinary tract, the external genitalia, and in the worse cases also the gastrointestinal tract. The BEEC represents the severe end of all human congenital anomalies of the kidney and urinary tract. The most common defect form, classic bladder exstrophy (CBE), is characterized by pubic diastasis, the evaginated bladder plate template, and an epispadic urethra. At birth, the visible bladder mucosa appears reddish and mucosal polyps may be seen on the surface. CBE is associated with kidney and other upper urinary tract anomalies with a higher occurrence in males compared to females^1^. Associated long-term complications include malignancies of the bladder comprising mainly urothelial cell carcinoma and adenocarcinoma^2,3^. Recently, the CBE live prevalence for Germany has been estimated to be ~1:30,700^4^. Given the overall European population of ~450,000,000 (https://ec.europa.eu/) citizens, presumptively ~15,000 CBE patients live in Europe. State-of-the-art health care for this population should take the genetic and bladder cancer disposition into account.

To determine the genetic contribution to CBE, we previously performed two genome-wide association studies (GWAS) with subsequent meta-analysis and identified a susceptibility locus on chromosome 5q11.1^5,6^. The present study aimed to identify further risk loci. Furthermore, we investigated if the identified genetic risk loci might be involved in the associated bladder cancer susceptibility. For this purpose, we performed the largest GWAS for CBE to date comprising 628 patients and 7352 ethnically matched controls. In detail, the present meta-analysis included seven independent discovery samples (Supplementary Information: Supplementary Table 1) comprising: 98 patients of Central European origin and 526 ethnically matched controls^5^, 110 patients of Central European origin and 1,177 ethnically matched controls^6^, 172 patients of Central European origin and 2588 ethnically matched controls, 57 patients of Italian origin and 1,325 ethnically matched controls, 62 patients of Spanish origin and 279 ethnically matched controls, 80 patients of Swedish origin and 238 ethnically matched controls, and 49 patients of UK origin and 1,219 ethnically matched controls, identifying eight genome-wide significant risk loci, seven of which are novel. Within these loci reside 10 coding genes (*LPHN2*, *EFNA1*, *SLC50A1*, *DPM3*, *KRTCAP2*, *ISL1*, *TRIM29*, *SYT1*, *PAWR*, *GOSR2*) and four non-coding genes (one pseudogene and three long non-coding RNA, respectively, HMGB1P47, ISL1-DT, LINC01974, and LINC01716). Among these coding genes, *EFNA1* has been previously shown to be strongly expressed in mouse embryonic genital tubercle, urethra, and primitive bladder prompting us to re-sequence this gene in our cohort. To assess their embryonic and fetal expression, we generated mouse embryonic bladder total RNA-seq at CBE-relevant developmental stages E10.5, E12.5, and E15.5, and human embryonic and fetal urinary bladder and genital tissues total RNA-seq at gestational week 7, 7 to 7.5, 7.5, 8, and 9. Finally, to evaluate their possible link in the overall CBE bladder cancer susceptibility, we analyzed the expression of these genes in urothelial carcinoma tissues and in different bladder cancer cell lines obtained from the Cancer Cell Line Encyclopedia (EMBL-EBI) compared to healthy bladder tissue transcriptomic (GEO).

## Results

### GWAS meta-analysis

**Fig. 1:**
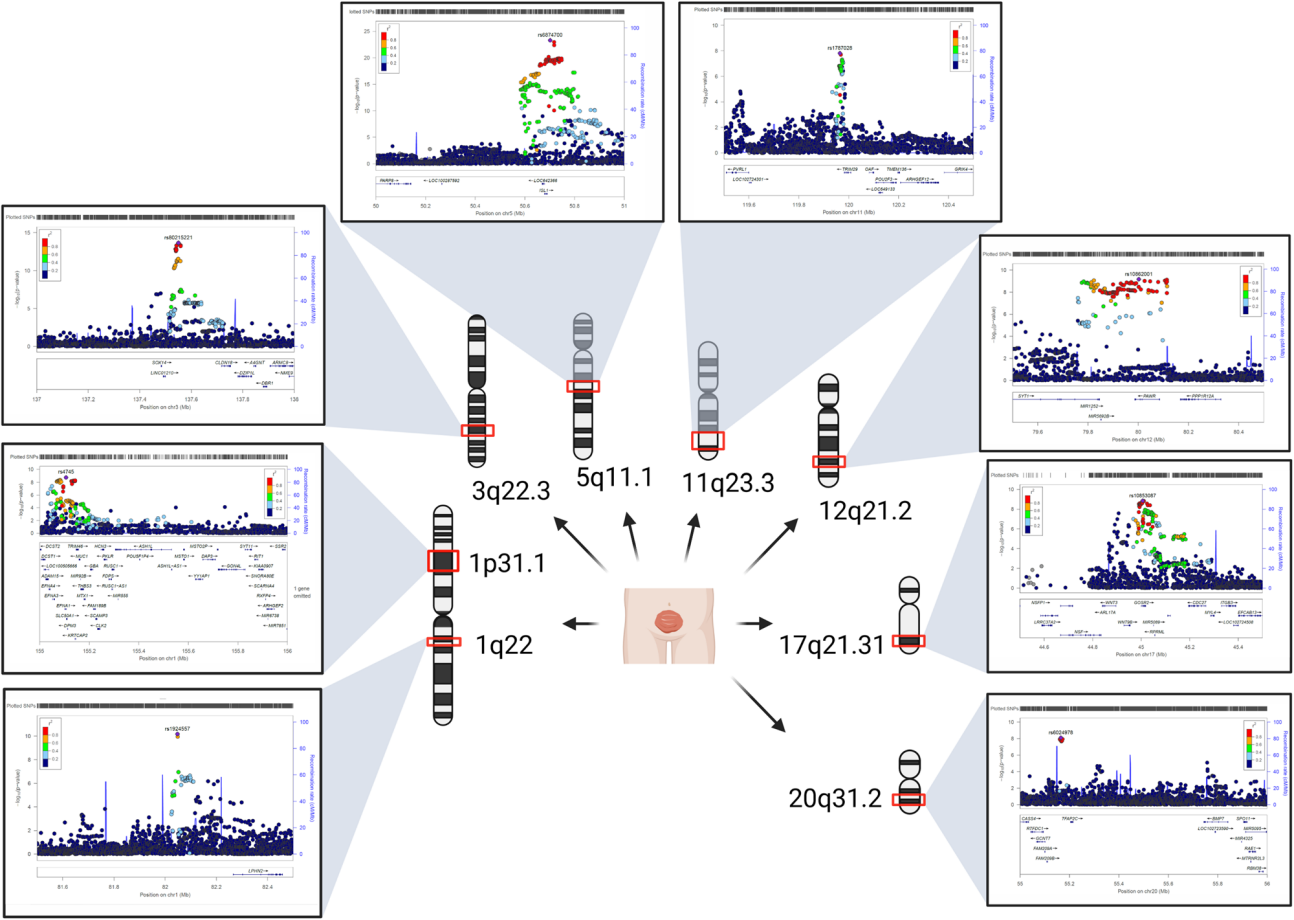
Chromosome regional association loci of CBE. The eight CBE regional association loci (red boxes in chromosomes) reside in chromosome 1, 3, 5, 11, 12, 17, and 20. In the panels, details of the genome-wide association loci: every dot represents an SNP (*x* axes) plotted to the relative –log_10_(*P* value) (*y* axes). SNP are colored according to the relative *r*^2^ value.

The meta-analysis of 628 patients with CBE and 7,352 ethnically matched controls comprised seven independent GWAS. These seven GWAS included the first two GWAS cohorts^5,6^, and five new CBE cohorts described above from Central European, Italy, Spain, Sweden, and the UK along with ethnically matched control samples. We used a total of 8,289,003 SNPs with info score >0.4 and mean dosage for the minor allele >1% in cases and controls in at least one sample, obtaining a genomic inflation factor λ of 1.068. The respective Q-Q plot is shown in Supplementary information (Supplementary Fig. 16). Single marker analysis identified eight genome-wide significant loci shown in the Manhattan plot in Supplementary information (Supplementary Fig. 17) and the strongest signal at rs6874700 *p* = 5.58 × 10^−24^ corresponds to the 5q11.1 previously reported locus (Fig. 1)^6^. Table 1 shows the relative risks in each sample and in the meta-analysis for the most strongly associated SNP (top SNP) from each locus. Notably, with the exception of the UK sample where the top associated SNP on chromosome 12 was not significant, the direction of effect was consistent between all studies for these top SNPs. A complete list of all genome-wide significant SNPs is given in Supplementary Data 2. Regional association results for all eight genome-wide significant loci are shown in Fig. 1 and in Supplementary Information (Supplementary Figs. 1–8). For conditional logistic regression analyses the regional association plots are presented in Supplementary Information (Supplementary Figs. 20–27). The results provide no evidence that secondary signals in any of the eight loci are present.

**Table 1:** Top SNPs from genome-wide significant loci.

				GWAS1	GWAS2	CE	Italy	Spain	Sweden	UK	Meta-analysis		
SNP	Chromosome	risk/other allele	Info Score^1^	RR (95% CI)	RR (95% CI)	RR (95% CI)	RR (95% CI)	RR 95% CI	RR 95% CI	RR 95% CI	RR 95% CI	*p* value	Corrected *p* value^2^
rs1924557	1:82047934	T/C	0.93	1.75 (0.68−4.47)	3.61 (1.37−9.49)	3.76 (2.16−6.53)	2.03 (0.98−4.19)	3.51 (1.33−9.26)	1.23 (0.68−2.22)	5.00 (1.59−15.7)	2.35 (1.82−3.03)	6.65 × 10^−11^	2.67 × 10^*−*10^
rs4745	1:155106227	T/A	0.96	1.19 (0.72−1.98)	1.40 (0.91−2.16)	1.47 (1.16−1.87)	1.80 (1.16−2.78)	1.17 (0.73−1.86)	1.63 (1.13−2.36)	1.16 (0.77−1.74)	1.50 (1.32−1.71)	4.44 × 10^−10^	1.58 × 10^−9^
rs80215221	3:137550420	G/A	0.99	2.29 (1.29−4.09)	1.95 (1.20−3.17)	1.39 (1.08−1.79)	1.89 (1.24−2.89)	2.82(1.76−4.52)	1.70 (1.16−2.51)	1.99 (1.31−3.03)	1.68 (1.47−1.92)	2.26 × 10^−14^	1.49 × 10^−13^
rs6874700	5:50701750	A/T	0.99	2.54 (1.49−4.33)	1.33 (0.87−2.02)	1.60 (1.28−2.01)	1.98 (1.33−2.95)	2.14 (1.33−3.45)	1.94 (1.32−2.83)	1.84 (1.20−2.80)	1.91 (1.68−2.16)	5.58 × 10^−24^	1.48 × 10^*−*22^
11:119964758:G:C	11:119964758	G/C	0.98	1.38 (0.81−2.36)	1.54 (0.98−2.42)	1.32 (1.03−1.69)	1.35 (0.90−2.04)	1.14(0.72−1.80)	1.68 (1.12−2.52)	2.13 (1.40−3.24)	1.47 (1.29−1.68)	8.89 × 10^−9^	2.63 × 10^*−*8^
rs10862001	12:80002106	A/G	0.83	2.02 (1.12−3.64)	1.48 (0.89−2.47)	1.77 (1.27−2.45)	1.97 (1.22−3.18)	1.03 (0.55−1.93)	2.11 (1.26−3.54)	0.83 (0.40−1.69)	1.68 (1.43−1.99)	6.84 × 10^−10^	2.37 × 10^*−*9^
rs10853087	17:45006112	C/G	0.97	1.18 (0.72−1.93)	1.44 (0.94−2.21)	1.63 (1.29−2.06)	1.60 (1.08−2.38)	1.46 (0.93−2.29)	1.26 (0.87−1.83)	1.53 (1.01−2.31)	1.47 (1.30−1.66)	1.41 × 10^−9^	4.67 × 10^*−*9^
20:55165923:G:A	20:55165923	G/A	0.97	2.30 (1.13−4.67)	1.72 (0.95−3.12)	1.46 (1.08−1.98)	2.57 (1.39−4.76)	1.00 (0.58−1.71)	2.08 (1.23−3.51)	1.89 (1.04−3.43)	1.65 (1.39−1.96)	7.31 × 10^−9^	2.19 × 10^*−*8^

^1^Mean imputation quality score (Info score)). Chromosomal positions are annotated according to Human Genome version 19 (hg19); *CE* Central Europe, *UK* United Kingdom

^2^*p* value corrected for genomic inflation *λ* = 1.068

### Re−sequencing of *EFNA1*

Among the most significant markers, marker rs4745 resides directly in *EFNA1*. Mouse *Efna1* has been shown to be expressed in CBE-relevant embryonic anatomical structures (https://www.gudmap.org/). This prompted us to re-sequence *EFNA1* in 580 CBE patients. We identified 14 rare variants in 14 independent patients (Supplementary information, Supplementary Table 4). Four of these variants residing in the coding region of *EFNA1* were found to be novel: two heterozygous missense variants c.116 T > C (p.Ile39Thr) and c.503 C > T (p.Ala168Val); one homozygous missense variant c.167 A > G (p.Asp56Gly), and a heterozygous loss of function (LoF) frameshift variant at c.341delT (p.Phe114Serfs*28). Parental samples were only available for the patient carrying variant c.116 T > C demonstrating paternal transmission. In silico prediction tools, Mutation Taster, Poly-Phen-2, and SIFT defined the missense variant c.116 T > C (p.Ile39Thr) as disease-causing, deleterious, and possibly damaging. The CADD score of 25.3 supports a functional implication of this variant on *EFNA1* regulation. None, of the other missense variants, were scored deleterious. The LoF variant c.341delT (p.Phe114Serfs*28) has a CADD score of 25.6. For the estimation of the enrichment of rare protein-altering variants in EFNA1 in our cohort compared to the general population resembled by gnomAD, we use a very conservative comparison. Hence, we only included the three novel coding variants with CADD score >20, identified here in our re-sequencing approach of *EFNA1*. We compared these to missense or LoF variants in gnomAD less or equal to 5 (≤5 in 250,000; MAF ≤ 0,00002) consistent with rare penetrant dominant phenotypes. These criteria identified 162 missense and LoF variants in gnomAD (baseline 250,000 alleles; https://gnomad.broadinstitute.org/gene/ENSG00000169242?dataset=gnomad_r2_1). Per se, it is possible that some of these variants are cis/trans in the same individuals but if we would be able to define this it would only make the association stronger. For comparison, we used Fisher’s exact test. Taking this assumption, the chi-square statistic using Fisher´s exact test, yielded 18.0159, and the *p* value is 0.000022. We added this statistic to our results.

### Analysis of mouse and human embryonic total RNA-sequencing data of the identified genes in mouse and human embryonic and fetal urinary bladder and genital tissues

**Fig. 2:**
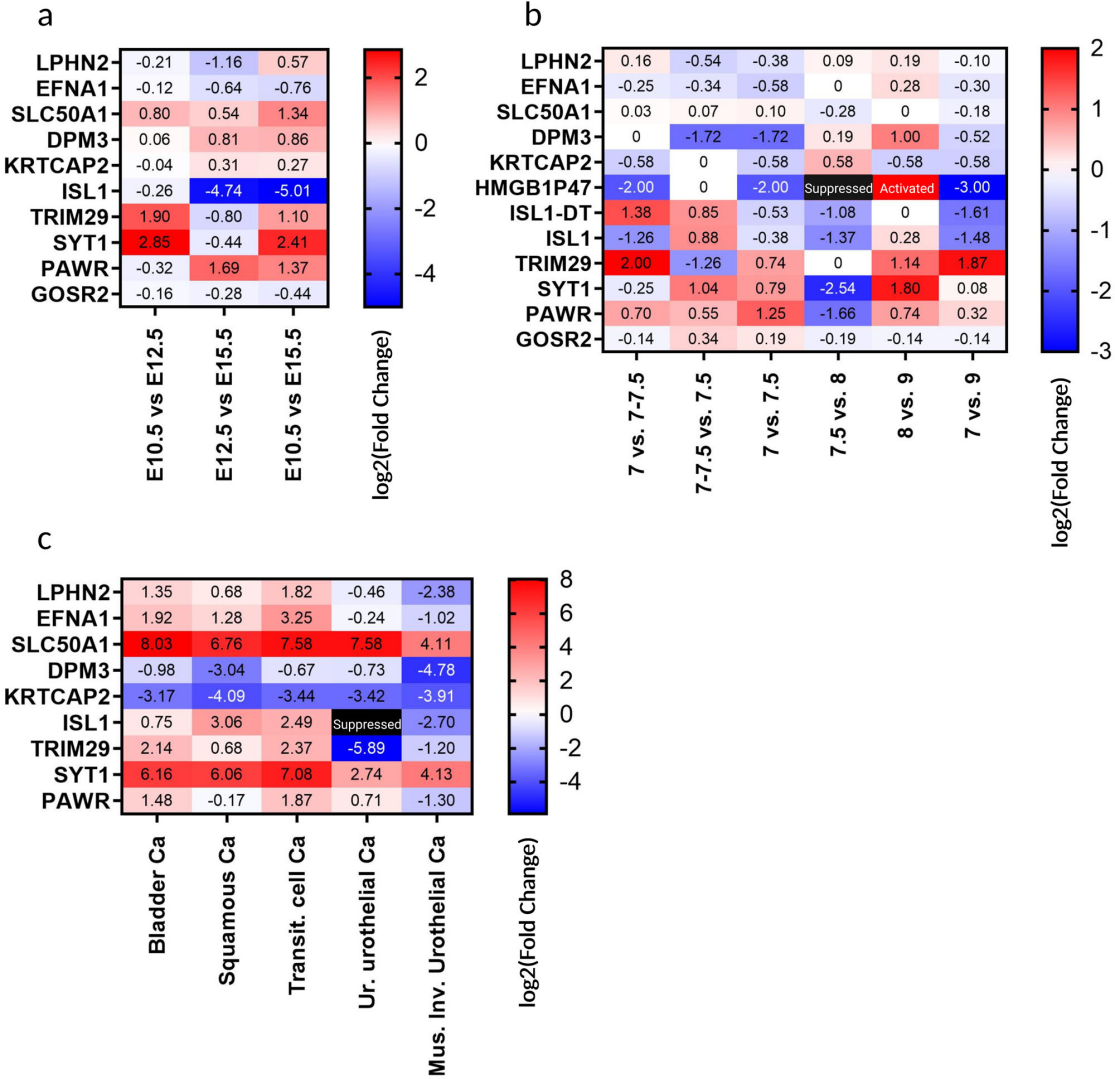
Expression heatmaps of genes that reside in the LD blocks of the eight significant genetic markers. **a** Genes expression pattern in mouse embryonic bladder from embryonic day E10.5 to E12.5, E12.5 to E15.5, and E10.5 to E15.5. **b** Genes expression pattern in human embryonic and fetal bladder from week 7 to 7–7.5, 7–7.5 to 7.5, 7 to 7.5, 7.5 to 8, 8 to 9, and 7 to 9. **c** Genes expression pattern of 3-year-old control bladder tissue compared to Bladder carcinoma (Bladder Ca), Bladder squamous cell carcinoma (Squamous cell Ca), Bladder transitional cell carcinoma (Transit. cell Ca), Ureter urothelial carcinoma (Ur. Urothelial Ca), Muscle invasive urothelial cancer (Mus. Inv. Urothelial Ca). Legend: Suppressed = gene is silenced and no expression is detected. Activated = gene shows expression after a silenced state.

In the linkage disequilibrium block of all eight top SNPs reside 10 coding genes, four non-coding genes comprising one pseudogene, and three Long Intergenic Non-Protein Coding RNAs (Table 2). All 10 coding genes showed expression in mouse embryonic bladder at E10.5, E12.5, and E15.5. *Isl1*, *Trim29*, *Syt1*, and *Pawr* showed differential expression through different mouse embryonic stages (Table 3, Fig. 2a). As in mouse transcriptome, all 10 coding genes showed expression in the human embryonic bladder during different developmental stages. *DPM3*, *TRIM29*, *SYT1,* and *PAWR* showed differential expression at different human embryonic respectively fetal stages (Table 3, Fig. 2b). While the two *LINC01974* and *LINC01716* are not expressed in any of the bladder developmental stages, the pseudogene *HMGB1P45* and the long noncoding RNA *ISL1-DT* are strongly downregulated; the first from weeks 7 to 7.5 followed by gene silencing at week 8 and 9 and the latter shows a downregulation trend from weeks 7 to 9.

**Table 2:** Coding and non-coding genes in the LD blocks of the most significant markers for CBE.

Gene name	Associated SNP	Protein encoded	Type	Cellular location	Function
LPHN2	rs1924557	Adhesion G protein-coupled receptor L2	G protein-coupled receptor	Transmembrane of plasma membrane	Exocytosis regulator
EFNA1	rs4745	Ephrin-A1	Tyrosine kinase receptor	Transmembrane of plasma membrane	GPI-bound ligand for Eph receptors, involved in cell migration, repulsion, and adhesion
SLC50A1	rs4745	Solute Carrier Family 50 Member 1	Glucose transporter	Transmembrane of plasma membrane	Sugar transport across membranes
DPM3	rs4745	Dolichol-phosphate mannosyltransferase subunit 3	Synthase of mannosyl residual	Endoplasmic reticulum membrane	Stabilizer subunit of DPM complex (DPM1, DPM2, and DPM3)
KRTCAP2	rs4745	Kerotinocyte-associated protein 2	Subunit of the oligosaccharyl transferase complex	Endoplasmic reticulum membrane	Protein N-glicosilation. Transfer of defined glycan (Glc(3)Man(9)GlcNAc(2).
ISL1	rs6874700	Insulin Gene Enhancer Protein ISL-1	Transcription Factor	Nucleus	DNA-binding transcriptional activator
TRIM29	11:119964758	Tripartite motif-containing protein 29	Zinc finger and Leucine zipper motif		Nucleic acid binding and macrophage activation
SYT1	rs10862001	Synaptotagmin-1	Ca(2+) sensor	Transmembrane of synaptic vesicles	Triggering neurotransmitter release
PAWR	rs10862001	Pro-Apoptotic WT1 Regulator	Apoptosis inducer	Nucleus and cytoplasm	Downregulation of BCL2 via its interaction with WT1
GOSR2	rs10853087	Golgi SNAP receptor complex member 2	Trafficking membrane protein	Golgi	Protein transport from the cis/medial-Golgi
LINC01974	rs10853087	/	Long ncRNA	Unknown	Unknown
LINC01716	20:55165923	/	Long ncRNA	Unknown	Unknown
HMGB1P47	rs6874700	/	Long ncRNA	Unknown	Unknown
ISL1-DT	rs6874700	/	Long ncRNA. ISL1 divergent transcript.	Unknown	Unknown

**Table 3:** RNA expression patterns of coding and non-coding genes in the LD blocks of the most significant GWAS markers in mouse and human embryonic urogenital tissue.

Mouse embryonic transcriptome data					Human embryonic transcriptome data						
Marker	Gene	log2fc E10.5 vs. E12.5	log2fc E10.5 vs. 15.5	log2fc E12.5 vs. 15.5	Gene	log2fc 7 vs. 7-7.5	log2fc 7-7.5 vs. 7.5	log2fc 7 vs. 7.5	log2fc 7.5 vs. 8	log2fc 7 vs. 9	log2fc 8 vs. 9
rs1924557	LPHN2	−0.21	0.57	−1.16	LPHN2	0.16	−0.54	−0.38	0.09	−0.10	0.19
rs4745	EFNA1	−0.12	−0.76	−0.64	EFNA1	−0.25	−0.34	−0.58	0.00	−0.30	0.28
rs4745	SLC50A1	0.80	1.34	0.54	SLC50A1	0.03	0.07	0.10	−0.28	−0.18	0.00
rs4745	DPM3	0.06	0.86	0.81	**DPM3**	0.00	**−1.72**	**−1.72**	0.19	−0.52	1.00
rs4745	KRTCAP2	0.27	0.31	−0.04	KRTCAP2	−0.58	0.00	−0.58	0.58	−0.58	0.58
rs6874700	**ISL1**	−0.26	**−5.01**	**−4.76**	ISL1	−1.26	0.88	−0.38	−1.37	−1.48	0.28
11:119964758	**TRIM29**	**1.90**	1.10	−0.80	**TRIM29**	**2.00**	−1.26	0.74	0.00	**1.87**	1.14
rs10862001	**SYT1**	**2.85**	**2.41**	−0.44	**SYT1**	−0.25	1.04	0.79	**−2.54**	0.08	**1.8**
rs10862001	**PAWR**	−0.32	1.37	**1.69**	**PAWR**	0.70	0.55	1.25	**−1.66**	0.32	0.74
rs10853087	GOSR2	−0.16	−0.44	−0.28	GOSR2	−0.14	0.34	0.19	−0.19	−0.14	−0.14

Marker	Non-coding Gene	log2fc E10.5 vs. E12.5	log2fc E10.5 vs. 15.5	log2fc E12.5 vs. 15.5	Non-coding gene	log2fc 7 vs. 7–7.5	log2fc 7–7.5 vs. 7.5	log2fc 7 vs. 7.5	log2fc 7.5 vs. 8	log2fc 7 vs. 9	log2fc 8 vs. 9
rs6874700	**HMGB1P47**	n.e.			**HMGB1P47**	**−2.00**	0.00	**−2.00**	activated	**−3.00**	suppressed
rs6874700	**ISL1−DT**	n.e.			**ISL1−DT**	−1.38	0.85	−0.53	−1.8	**−1.61**	0.00
rs10853087	LINC01974	n.e.			LINC01974			n.e.			
20:55165923	LINC01716	n.e.			LINC01716			n.e.			

Bold: differential expressed genes (log2fc <−1.5 or >1.5).

*log2fc* log2 fold change, *vs*. versus, *Chr*. chromosome, *n.e*. not expressed. Differential expression defined with log2fc <−1,5 or >1,5.

### Comparison of RNA-sequencing data of the identified genes in healthy bladder tissue and different types of bladder cancer cell lines and muscular invasive urothelial carcinoma

Expression of non-coding genes in healthy bladder tissue and muscular invasive urothelial carcinoma could not be detected due to polyA-RNA-sequencing (Table 4). Despite this, we found all coding genes to be differentially expressed when compared to healthy bladder tissue (Table 5, Fig. 2c). In detail, *SLC50A1* and *SYT1* were significantly upregulated in all cancers compared to healthy bladder tissue. On contrary, *DPM3* and *KRTCAP2* were significantly downregulated. All other coding genes *LPHN2*, *EFNA1*, *ISL1*, *TRIM29*, *PAWR*, and *GOSR2* were differentially up- or downregulated in different cancers (Table 5, Fig. 2c).

**Table 4:** Average TPM of coding and non-coding genes in the LD blocks of the most significant GWAS markers calculated for each bladder cancer cell line.

Marker	Coding and non-coding genes	Control tissue	Bladder Ca	Transit. cell Ca	Squamous cell Ca	Ur. urothelial Ca	Mus. Inv. Urothelial Ca
rs1924557	LPHN2	6.87	17.47	24.25	11.00	5.00	1.32
rs4745	EFNA1	8.25	31.30	78.50	20.00	7.00	4.06
rs4745	SLC50A1	0.24	62.60	46.00	26.00	46.00	4.15
rs4745	DPM3	131.29	66.55	82.50	16.00	79.00	4.77
rs4745	KRTCAP2	170.89	18.95	15.75	10.00	16.00	11.37
rs6874700	ISL1	0.36	0.61	2.03	3.00	0.00	0.06
11:119964758	TRIM29	11.86	52.27	61.25	19.00	0.20	5.15
rs10862001	SYT1	0.06	4.30	8.13	4.00	0.40	1.05
rs10862001	PAWR	13.47	37.65	49.25	12.00	22.00	5.46
rs10853087	GOSR2	11.25	16.80	12.50	5.00	16.00	3.69
rs6874700	HMGB1P47	RNA-polyA-seq	0.00	0.00	0.00	0.00	RNA-polyA-seq
rs6874700	ISL1-DT	RNA-polyA-seq	0.11	0.20	0.5	0.00	RNA-polyA-seq
rs10853087	LINC01974	RNA-polyA-seq	0.04	0.00	0.00	0.00	RNA-polyA-seq
20:55165923	LINC01716	RNA-polyA-seq	0.01	0.00	0.00	0.00	RNA-polyA-seq

urothelial carcinoma tissues and control bladder tissue.

TPM for Non-coding genes in Control tissue and Muscolar invasive urothelial cancer do not show reads due to polyA-sequencing. Legend: Bladder tissue of a 3-year-old bladder donor (control tissue); Bladder carcinoma (Bladder Ca); Bladder transitional cell carcinoma (Transit. cell Ca); Bladder squamous cell carcinoma (Squamous Ca); Ureter urothelial carcinoma (Urothelial Ca); Bladder urothelial carcinoma (Bl. urothelial Ca); Muscle invasive urothelial cancer (Mus. Inv. Urothelial Ca); RNA-polyA-seq data do not include miRNAs or lincRNAs.

**Table 5:** log2-fold change of genes in the LD blocks of the most significant GWAS markers of bladder cancer cell types and muscle-invasive urothelial cancer over bladder control tissue.

Marker	Coding and non-coding genes	Control tissue vs. bladder Ca	Control tissue vs. transit. cell Ca	Control tissue vs. squamous cell Ca	Control tissue vs. Ur. urothelial Ca	Control tissue vs. Mus. Inv. urothelial Ca
rs1924557	**LPHN2**	1.35	**1.82**	0.68	0.46	**−2.38**
rs4745	**EFNA1**	**1.92**	**3.25**	1.28	−0.24	−1.02
rs4745	SLC50A1	**8.03**	**7.58**	**6.76**	**7.58**	**4.11**
rs4745	**DPM3**	−0.98	−0.67	**−3.04**	−0.73	**−4.78**
Rs4745	**KRTCAP2**	**−3.17**	**−3.44**	**−4.09**	**−3.42**	**−3.91**
rs6874700	**ISL1**	0.75	**2.49**	**3.06**	suppressed	**−2.70**
11:119964758	**TRIM29**	**2.14**	**2.37**	0.68	**−5.89**	−1.20
rs10862001	**SYT1**	**6.16**	**7.08**	**6.06**	**2.74**	**4.13**
rs10862001	**PAWR**	1.48	**1.87**	−0.17	0.71	−1.30
rs10853087	**GOSR2**	0.54	0.12	−1.21	−0.47	**−1.65**
rs6874700	HMGB1P47	RNA-polyA-seq	RNA-polyA-seq	RNA-polyA-seq	RNA-polyA-seq	RNA-polyA-seq
rs6874700	ISL1-DT	RNA-polyA-seq	RNA-polyA-seq	RNA-polyA-seq	RNA-polyA-seq	RNA-polyA-seq
rs10853087	LINC01974	RNA-polyA-seq	RNA-polyA-seq	RNA-polyA-seq	RNA-polyA-seq	RNA-polyA-seq
20:55165923	LINC01716	RNA-polyA-seq	RNA-polyA-seq	RNA-polyA-seq	RNA-polyA-seq	RNA-polyA-seq

Bold: differential expressed genes (log2fc <−1.5 or >1.5)

Log2 fold change for non-coding genes of different bladder cancers and Muscolar Invasive Urothelial cancer over control tissue shows no value due to polyA-sequencing of control tissue. Differential Expression defined with log2fc <−1.5 or >1.5 (marker in bold italic letters). Legend: Bladder tissue of a 3-year-old bladder donor (control tissue); Bladder carcinoma (Bladder Ca); Bladder transitional cell carcinoma (Transit. cell Ca); Bladder squamous cell carcinoma (Squamous Ca); Ureter urothelial carcinoma (Urothelial Ca); Bladder urothelial carcinoma (Bl. urothelial Ca); Muscle invasive urothelial cancer (Mus. Inv. Urothelial Ca); RNA-polyA-seq data does not include miRNAs or lincRNAs). *log2fc* log2 fold change; differential expression defined with log2fc <−1.5 or >1.5 (marker in bold italic letters). *vs*. versus, *Chr*. chromosome.

## Discussion

Recently, we described *SLC20A1*, encoding a sodium-phosphate symporter, as the putative monogenic dominant disease gene for isolated BEEC^7^. We were able to support our genetic data through functional studies in non-BEEC human embryos, mouse embryos, and zebrafish Morpholino knockdown experiments. To our knowledge, the present genetic study with a focus on the multifactorial genetic background of the BEEC is the largest study on CBE to date. We identified eight genome-wide significant risk loci. Within these loci, we determined possible CBE candidate genes using transcriptome datasets of CBE-relevant mouse embryonic, human embryonic, and fetal urogenital tissues at different developmental stages. Additionally, we provide a possible link between the identified putative candidate genes and CBE-associated bladder cancer susceptibility.

In detail, in direct proximity to the most significant markers of all eight risk loci reside nine coding genes that are expressed in CBE-relevant mouse and human urogenital tissues during different embryonic stages. Four of these candidate genes (*Isl1*, *Trim29*, *Syt1*, *Pawr*) showed differential expression in mouse embryonic urogenital tissues, five of these candidate genes (*DPM3*, *ISL1*, *TRIM29*, *SYT1*, and *PAWR*), and two of the non-coding genes (*HMGB1P47*, *ISL1-DT*) showed differential expression in human embryonic urogenital tissues. Previous reports of transgenic mouse lines of *Isl1* and *Syt1* revealed phenotypic overlap to the human CBE phenotypic spectrum. The *Hoxb6Cre*;*Isl1* cKO hindlimb skeletons exhibited proximal defects in particular the os pubis and ischium, two posterior segments of the pelvic girdle, were missing, resembling pubic diastasis, a human BEEC-specific feature^1,8^. Transgenic Syt1^tm1a(EUCOMM)Wtsi^/Syt1^tm1a(EUCOMM)Wtsi^ mice among other features develop thoracoschisis^9^, a rare congenital anomaly characterized by the evisceration of intra-abdominal organs through a thoracic wall defect^10^ mirroring the BEEC associated infraumbilical abdominal wall defect^1^.

One of the most significant markers identified in the present GWAS resides within *EFNA1*. In general, a probability of being LoF intolerant (pLi) score of 0.46 for *EFNA1* is suggestive of possessing LoF intolerance for this gene in the context of the CBE condition. Although the pLi of 0.46 is only suggestive of LoF we have to consider that bladder exstrophy is not a mortal condition at birth. Hence, we believe that a value of 0.46 is supportive for *EFNA1* to be implied in CBE^11^. Previously, in *EFNA1* only two LoF variants were observed in the entire gnomad (frequency of 0,000016). Here, we observed one in 580 (frequency of 0,0017). Furthermore, in *EFNA1* in the entire gnomad database, 96 missense variants were observed in 125,099 individuals (frequency of 0,00077). Here, we identified three in 580 (0,0052). Based on this observation, we performed a conservative estimation of whether LoF or missense variants might be enriched in *EFNA1* in our CBE cohort compared to the general population showing a significant difference between both cohorts (*p* 0.000022). This finding suggests a possible implication of these variants in CBE formation in a multifactorial inheritance model among the affected.

Comparative analysis of control and bladder cancer tissues showed that all of the ten candidate genes were differentially expressed in bladder cancers. *SLC50A1* and *SYT1* were significantly upregulated in all cancers compared to healthy bladder tissue. On contrary, *DPM3* and *KRTCAP2* were significantly downregulated. *LPHN2* has been suggested to have a regulatory role in urothelial bladder cancer^12^. *EFNA1* plays a pivotal role in the pathogenesis of several tumors, including renal cell carcinoma, bladder, and prostate cancer^13,14^. Mapping all putative candidate genes prioritized in the present study to the search tool for retrieval of interacting genes (STRING), we found probable interaction of three proteins comprising: (i) gene fusions between EFNA1 and SLC50A1, and (ii) co-expression between EFNA1 and DPM3, (iii) and EFNA1 and SLC50A1 (Supplementary information: Supplementary Fig. 28). The PPI enrichment p-value was determined with 0.000205. Gene clustering analysis suggested clustering for *EFNA1*, *DPM3*, and *SLC50A1* (Supplementary information: Supplementary Fig. 28). All three genes *EFNA1*, *DPM3*, and *SLC50A1*, respectively the genomic region 1q21-q22, been previously associated with the 2D:4D ratio, a sexually dimorphic trait, that has been extensively used in adults as a biomarker for prenatal androgen exposure^15^. Markers in the region of *EFNA1*, *DPM3, SLC50A1* have previously been associated with prostate cancer risk^16^. Prostate cancer risk on the other side correlates with serum testosterone levels^17^. All of these observations suggest a possible gene-environmental interaction for this region. Adding to this hypothesis in the context of embryonic CBE formation, CBE presents with a higher occurrence rate in males compared to females^1^, a skewed sex ratio that is so far not explained, but could be influenced by differences in intrauterine androgen exposure between males and females.

The tumor suppressor gene *TRIM29* is up regulated during early and late embryonic bladder development but is down-regulated in three different bladder cancers^18^. More specific, TRIM29 protein has been shown to be a driver of invasive and non-invasive bladder cancer. Interestingly, TRIM29-driven bladder cancers in transgenic mice were indistinguishable from gene expression signatures of human bladder cancers^19^. *PAWR*, has been previously shown to be a key altered gene in human bladder cancer stem cells^20^. *SYT1* has been reported as a possible oncogene in colon cancer^21^. The knockdown of *SYT1* markedly inhibits colon cancer cell proliferation, migration, and invasion, and induces cell apoptosis, indicating that *SYT1* may function as an oncogene in colon cancer^21^. *ISL1* has been associated with high-risk non-muscle-invasive bladder cancer in several studies^22,23^. Here we found downregulation of *ISL1* in embryonic stages of mouse and human CBE urogenital tissues. Vice versa we found dysregulation of *ISL1* expression in three bladder cancers. Hence, dysregulation of *ISL1* expression in human embryonic and adult bladder tissues might contribute to the CBE and bladder malignancies vice versa.

To date, this is the largest genetic study on CBE. We have identified eight genome-wide significant risk loci. Our transcriptomic analysis of CBE-relevant mouse embryonic, human embryonic, and fetal urogenital tissues suggests candidate genes within these loci. Bladder cancer transcriptomic suggests these candidate genes play a possible role in the CBE-associated bladder cancer susceptibility. Identification of the different expressions to turn these developmental genes on later in life might ultimately lead to preventive strategies for bladder cancer per se.

## Methods

### Patients and recruitment

This study was approved by the institutional ethics committee of each participating center. All experimental protocols were approved by the institutional committee of the University of Bonn (Lfd.Nr.031/19). The study was conducted according to the Declaration of Helsinki principles. Written informed consent was obtained from all patients, guardians, and healthy controls. We included 420 newly recruited isolated CBE patients and 5,649 healthy controls of European origin. Details can be found in the Supplementary information (Supplementary Table 1). Details about the 208 CBE patients and 1,703 ethnically matched controls of our previous studies, included in the present meta-analysis, are described in [5,6], in summary, CBE patients were recruited under written informed consent by BEEC expert physicians.

### Sample description

In addition to the two previously described samples GWAS1 and GWAS2^6^, five new samples of patients with bladder exstrophy and representative controls were obtained from Central Europe, the United Kingdom, Italy, Spain, and Sweden. The number of cases and controls used in this study are shown in Supplementary Information (Supplementary Table 1).

### Genotyping

All samples, cases, and controls, were genotyped on Illumina human genotyping arrays. In GWAS 1 (Reutter et al. 2014), cases and controls were genotyped in two batches. Due to the discontinuation of the genotyping arrays utilized for earlier batches, different arrays were used comprising Illumina’s Human610-Quad (H610Q) and Human660W-Quad Bead Chips and the Illumina HumanOmni1-Quad-v1 Bead Chip. In GWAS 2^6^, all cases and controls were genotyped using the Illumina BeadChip HumanOmniExpress. The five novel GWAS case samples were newly genotyped simultaneously using the Illumina “Infinium Global Screening Array-24 v2.0”. The five novels ethnically matched control samples were also genotyped using the Illumina “Infinium Global Screening Array-24 v2.0”. However, the five novel control samples were not genotyped together with the five novel case samples but independently of each other.

### Quality control of individuals

An individual was excluded if (i) the call rate was <97%; (ii) the rate of autosomal heterozygosity deviates more than six standard deviations from the mean; (iii) the rate of X-chromosomal heterozygous genotypes was >2% for a supposed male individual or <10% for a supposed female individual. PLINK version 1.9 and KING were used to detect pairs of closely related individuals within and between samples^24,25^. From each pair of individuals with an estimated identity by descending probability >0.2 or kinship coefficient >0.0884, the individual with a higher rate of missing genotypes was discarded. Individuals being outliers in a multidimensional scaling analysis (MDS) were removed. The post-quality control sample sizes are presented in Supplementary Table 1.

### Principal component analysis

Individuals with:

1 $${\left(\frac{{c}_{1}-{m}_{1}}{{s}_{1}}\right)}^{2}+{\left(\frac{{c}_{2}-{m}_{2}}{{s}_{2}}\right)}^{2}\ge 81$$

were considered to be outliers. c_1_ and c_2_ denote the first two MDS coordinates of the individual and *m*_1_, *m*_2_ and *s*_1_ and *s*_2_ denote the mean and standard deviation, respectively, of the first two MDS coordinates in European HapMap individuals. For the five new samples (Central Europe, Italy, Spain, Sweden, and the UK), Supplementary Information (Supplementary Figs. 9–13) show the first two MDS coordinates for all genotyped individuals together with Asian and African HapMap individual. For the samples of GWAS1 and GWAS2, the MDS coordinates used in our previous studies are shown in Supplementary Information (Supplementary Figs. 14, 15).

### Quality control of variants and imputation

Separately in each of the different ethnicity samples, SNPs were removed if (i) the minor allele frequency was <1% in either cases or controls; (ii) the successful genotyping rate was >95% in either cases or controls; (ii) the *p* value for Hardy–Weinberg equilibrium was <10^−4^ in controls or <10^−6^ in cases. SNPs satisfying the quality filters were uploaded for imputation on the University of Michigan Imputation Server using the Haplotype Reference Consortium panel^26^.

### Statistical analysis for genome-wide association analysis

Association testing was performed by logistic regression using SNPTEST version 2.5.2 for the allele dosage and adjusted for the sample-specific top five MDS coordinates^27^. For each SNP, a meta-analysis with the fixed-effects inverse variance-weighting approach was conducted by including only those samples in which the info score was >0.4 and the mean dosage for the minor allele was >1% in cases and controls for the respective SNP. SNPs reaching a *p* value <5*10^−8^ in the meta-analysis are considered to be genome-wide significant. Q-Q and Manhattan plots for the meta-analysis were created by SAS^28^. Regional association plots for genome-wide significant loci were generated with LocusZoom^29^. To look for secondary signals of association in loci of genome-wide significance, logistic regression using SNPTEST conditioned on the most associated SNP in the locus was carried out.

### Protein–protein interaction networks analysis

Putative candidate genes within identified risk loci were mapped to the STRING to acquire protein–protein interaction (PPI) networks (https://string-db.org/). The search tool integrates both known and predicted PPIs. Here it was used to predict functional interactions of proteins^30,31^. Active interaction sources, including text mining, experiments, databases, and co-expression as well as species limited to “Homo sapiens” and an interaction score >0.4 were applied to construct the PPI networks. In the networks, the nodes correspond to the proteins and the edges represent the interactions. STRING was employed to seek potential interactions among putative candidate genes. Active interaction sources, including experimental repositories, computational prediction methods, and public text collections as well as species limited to “Homo sapiens” and a combined score >0.4, were applied.

### Re-sequencing of *EFNA1*

Re-sequencing of all coding exons of *EFNA1* of transcript ENST00000368407.3 was performed in 580 CBE patients, all of which were included in the current GWAS. PCR conditions can be obtained upon request, primer sequences are shown in Supplementary information (Supplementary Table 2). Sequencing files for patient, parent, and control DNA were added to databases created using PreGap4 software, with control DNA processed as the reference sequence.

### Genes prioritization

Lower p-value SNP of each associated region was imputed in LDproxy Tool (https://ldlink.nci.nih.gov/?tab=ldproxy) for European populations of CEU (Utah residents from north and west Europe); TSI Toscani in Italia; FIN Finnish in Finland; GBR British in England and Scotland; IBS Iberian population in Spain. Out of this, genes that reside in the linkage disequilibrium blocks defined from LD variants of r^2 above 0.8 to the top SNPs were taken into consideration for this study (Supplementary information, Supplementary Figs. 1–8). LD blocks coordinate regions imputed in hg19 are described in Supplementary Information (Supplementary Table 3).

No variants were significant LD associated with rs1924557 in chromosome 1 to determine an LD block region.

### RNA isolation and mRNA library preparation of mouse embryonic urinary bladder and genital tissues

Animals were anesthetized with Isoflurane and sacrificed by cervical dislocation. Ethical consent is documented and approved by the local authorities of the Regierungspräsidium Darmstadt. Embryos from timed-pregnant females of the SWISS strain were harvested at embryonic days (E) E10.5, E12.5, and 15.5 (Supplementary information: Supplementary Fig. 18). The respective developmental Theiler stages were determined as 18 (TS18), TS21, and TS23. From E10.5 embryos, the urogenital ridge was dissected under an M205C stereo microscope (Leica Microsystems, Germany) surgically isolated, and transferred into QIAzol®. Embryos were pooled for each time point. For E10.5 stage biopsies from three embryos were pooled biopsies to prepare RNA, for E12.5 and E15.5 stages two embryos were pooled for RNA preparation. From E12.5 (primitive bladder) and E15.5 (bladder) embryos, the distinct structures of the developing and distinct visible bladder were surgically isolated (Supplementary information: Supplementary Fig. 18), combined, and transferred into QIAzol®.

### Processing of total mouse embryonic RNA-sequencing data

About 50 million unique mapped reads per sample were obtained from each RNA-seq experiment. The reads were aligned using STAR aligner^32^. Read count was calculated with GenomicFeatures Bioconductor package. Calculation and normalization of “transcripts per kilobase million (TPM)” accounting for reads per kilobase (RPK) was performed as described elsewhere^33^. The fold change was calculated by dividing the subsequent stage by the preceding one and the log2 function was applied to the division as following: log2 (FoldChange) = log2 (subsequent embryonic stage/preceding embryonic stage). Differentially expressed genes were identified with values less than or equal to −1.5 or ≥1.5, respectively. The same algorithm was applied for the calculation of TPM of already deposited human embryonic and fetal RNA-seq data at EMBL-EBI expression atlas (accession code: E-MTAB-6592).

The raw RNA-sequencing data of mouse embryonic urinary bladder are deposited at GEO with the accession id: GSE190641.

### Processing of bladder cancer RNA-sequencing data

Total RNA was purified using the QuantSeq library (Lexogen) with 500 ng RNA input. QuantSeq polyA RNA-tail libraries were sequenced (Single end 1 × 75 bp) on an Illumina Hiseq platform and generated data were further processed according to the GRCh38, TPM transformed, and further normalized. Sequencing, aligning and TPM calculation was performed by ImmunityBio^TM^. Visualization of results in heatmaps was performed using graphpad PRISM 9.0.0.

### RNA isolation and mRNA library preparation of human embryonic and fetal urinary bladder and genital tissues

Embryonic and fetal bladders and genital tissues were obtained by surgeons from terminated pregnancies after informed consent was obtained and with ethics approval. Pregnancies were terminated for social indications and the respective fetuses and embryos were healthy. The embryonic tissues comprised 7-week embryos, 7–7.5-week embryos, 7.5-week embryos, late 8-week embryos, and late 9-week embryos (Supplementary information: Supplementary Fig. 19). Samples comprised week 7 (*n* = 2), 7.5 (*n* = 1), 8 (*n* = 3), 9 (*n* = 4) for the bladder tissues and for the genital tissues from week 7 (*n* = 3), 8 (*n* = 3), 9 (*n* = 3), and 10 (*n* = 4). Gene expression data were extracted and analyzed after high throughput sequencing of paired-end mRNA libraries (Illumina). Data were deposited at EMBL-EBI expression atlas (accession code: E-MTAB-6592). Calculation of fold change of already deposited human embryonic RNA-seq data was carried out accordingly to our calculation of mouse embryonic data (see in Methods: Processing of total mouse embryonic RNA-sequencing data).

### Processing of bladder cancer RNA-sequencing data

Cancer RNA-seq data were obtained from already deposited data at EMBL-EBI expression atlas (Cancer Cell Line Encyclopedia, experiment E-MTAB-2770). The deposited data does not include samples derived from CBE patients. Out of 1019 different cancer cell lines, the following cell line sample has been analyzed: 20 cell lines of bladder carcinoma, one cell line of bladder squamous cell carcinoma, four cell lines of bladder transitional cell carcinoma, one cell line of ureter urothelial carcinoma. TPM average was then calculated for each carcinoma cell type and data were compared with fold change to TPM of deposited mature urinary bladder polyA RNA-seq data (GEO accession: GSM1067793). In addition, RNA-polyA-seq data available from 38 cases of the CCC-EMN bladder cancer cohort [12] were generated from FFPE tissue all classified with Muscular invasive urothelial carcinoma. Demographic data is found in Supplementary Data 1.

### Statistics and reproducibility

Quality control of individuals, principal component analysis, Quality control of variants and imputation, Statistical analysis for GWAS, and Genes prioritization is meticulously described in the methods above.

Average of TPM was calculated in R from biological replicates. Log2FC of human and mouse bladder was calculated as following: log_2_(AVERAGE_TPM_next_stage_/AVERAGE_TPM_previous_stage_). Log2FC of cancer cells was calculated as following: log_2_(AVERAGE_TPM_cancer_line_/AVERAGE_TPM_control_bladder_tissue_). Sample size of mouse consists in *n* = 3 for embryo bladder at stage E10.5, and *n* = 2 for E12.5 and E15.5. Human fetal bladder samples comprised week 7 (*n* = 2), 7.5 (*n* = 1), 8 (*n* = 3), 9 (*n* = 4). Human cancer cells liens comprised *n* = 20 of bladder carcinoma cell, *n* = 1 of bladder squamous cell carcinoma cell line, *n* = 4 of bladder transitional cell carcinoma cell lines, *n* = 1 of ureter urothelial carcinoma cell line, *n* = 38 of muscular invasive urothelial carcinoma. Replicates are defined as a minimum of three technical replicates per sample size.

### Reporting summary

Further information on research design is available in the Nature Research Reporting Summary linked to this article.

## Supplementary information

Supplementary Information

Description of Additional Supplementary Data

Supplementary Data 1

Supplementary Data 2

Reporting Summary

## Data Availability

GWAS generated and analyzed data during this study are included in this article and its supplementary information files. GWAS data are deposited at NHGRI-EBI GWAS Catalog with accession ID: GCST90132313. EFNA1 DNA sequencing data are deposited in GeneBank (BankIt) with the following accession numbers: OP312051; OP312052; OP312053; OP312054; OP312055; OP312056; OP312057; OP312058; OP312059; OP312060; OP312061; OP312062; OP312063. The raw RNA-sequencing data of the 38 Muscular Invasive Urothelial carcinomas are deposited at NCBI in Sequence Read Archive (SRA) with the following BioProject accession: PRJNA882449. The raw RNA-sequencing data of mouse embryonic urinary bladder are deposited at GEO with the accession id: GSE190641. The raw RNA-sequencing data of human embryonic and fetal urinary bladder and genital tissue are deposited at EMBL-EBI expression atlas with the following accession id: E-MTAB-6592. The raw RNA-sequencing data of cancer cell lines are obtained from EMBL-EBI expression atlas with the following accession id: E-MTAB-2770, PolyA RNA-sequencing of the mature urinary bladder is obtained from GEO with the following accession id: GSM1067793.
