# Supplementary material for: A genome-wide association study with tissue transcriptomics identifies genetic drivers for classic bladder exstrophy
Source: Commun Biol. 2022 Nov 9;5:1203. doi: 10.1038/s42003-022-04092-3 (PMC9646906; doi:10.1038/s42003-022-04092-3)

Supplementary Information

A genome-wide association study with tissue transcriptomics identifies genetic drivers for classic bladder exstrophy

Authors Enrico Mingardo, Glenda Beaman, Philip Grote, Agneta Nordenskjöld, William Newman, Adrian S. Woolf, Markus Eckstein, Alina C. Hilger, Gabriel C. Dworschak,Wolfgang Rösch, Anne-Karolin Ebert, Raimund Stein, Alfredo Brusco, Massimo Di Grazia, Ali Tamer, Federico M. Torres, Jose L. Hernandez, Philipp Erben, Carlo Maj, Jose M. Olmos, Jose A. Riancho, Carmen Valero, Isabel C. Hostettler, Henry Houlden, David J. Werring, Johannes Schumacher, Jan Gehlen, Ann-Sophie Giel, Benedikt C. Buerfent, Samara Arkani, Elisabeth Åkesson, Emilia Rotstein, Michael Ludwig, Gundela Holmdahl, Elisa Giorgio, Alfredo Berettini, David Keene, Raimondo M. Cervellione, Nina Younsi, Melissa Ortlieb, Josef Oswald, Bernhard Haid, Martin Promm, Claudia Neissner, Karin Hirsch, Maximilian Stehr, Frank-Mattias Schäfer, Eberhard Schmiedeke, Thomas M. Boemers, Iris A. L. M. van Rooij, Wouter F. J. Feitz, Carlo L. M. Marcelis, Martin Lacher, Jana Nelson, Benno Ure, Caroline Fortmann, Daniel P. Gale, Melanie M. Y. Chan, Kerstin U. Ludwig, Markus M. Nöthen, Stefanie Heilmann, Nadine Zwink, Ekkehart Jenetzky, Benjamin Odermatt, Michael Knapp, Heiko Reutter

Supplementary Table 1. Number of cases and controls for the seven independent samples

Sample	No of Cases	No of Controls
**GWAS1**	98	526
**GWAS2**	110	1.177
**Central**	172	2.588
**Italy**	57	1.325
**Spain**	62	279
**Sweden**	80	238
**UK**	49	1.219

Supplementary Table 2. Primer sequences used for *EFAN1* re-sequencing

EFNA1 EX1 FWD	AAAGGCGGAGTCGCTAGG
EFNA1 EX1 REV	GGGGTGCTCCCAGATATGAC
EFNA1 EX2 FWD	CTTGGGGTCCAGTGTGAAAT
EFNA1 EX2 REV	GCTAAACAGAGTGCCCAGCA
EFNA1 EX3-4 FWD	GAGTAGGGAGCTGAGAAAGCA
EFNA1 EX3-4 REV	CTCTCAGCCCAACAGGATTC
EFNA1 EX5 FWD	AAGGGGTCTGCTTGAAGAGG
EFNA1 EX5 REV	CGTTTTGAGGCTGCTAGGTG

Legend: exon (EX); Forward (FWD); Reverse (REV).

Supplementary Table 3. Linkage Disequilibrium (LD) blocks coordinates (hg19) for CBE associated top variants.

Marker	Chromosome (chr)	chr Start	chr End
rs1924557	chr1	n.a.	n.a.
rs4745	chr1	155089883	155142927
rs80215221	chr3	137538620	137558922
rs6874700	chr5	50659788	50748173
rs1790471	chr11	119964758	119968219
rs10862001	chr12	79847040	80114135
rs10853087	chr17	44989888	45046865
rs6024978	chr20	55161209	55175996

Legend: n.a. = no LD association.

Supplementary table 4. Re-sequencing of EFNA1

Patients (n)	cDNA / protein change	exon / intron	SNP ID	gnomAD allele Frequency	Mutation Taster	PolyPhen-2	SIFT	CADD
n=1	93-36T>C	intron1	rs369393260	0.000256				
n=1	c.92+31G>A	intron1	rs372698388	0.00024				
n=1	c.92+117C>A	intron1	not reported	0				
n=1	c.92+147C>G	intron 1	not reported	0				
n=1	c.116T>C p.Ile39Thr	exon 2	not reported	0	disease causing	deleterious	possibly damaging	25.3
n=1	c.156G>A p.Pro52=	exon 2	rs376532577	0.000028	=	=	=	21.5
n=1	c.167A>G p.Asp56Gly	exon 2	not reported	0	disease causing	tolerated	benign	22.0
n=1	c.341delT p.Phe114Serfs*28	exon 2	not reported	0	LoF	LoF	LoF	25.6
n=1	c.455-13T>C	intron3	not reported	0				
n=1	c.454+75A>G	intron3	rs1033536381	6.6×10^{-06}				
n=1	c.454+61A>T	intron3	not reported	0				
n=1	c.455-52C>T	intron 3	not reported	0				
n=1	c.503C>T p.Ala168Val	exon 4	not reported	0	disease causing	tolerated	benign	6.97
n=1	c.521G>A p.Arg174Gln	exon 5	rs139969988	0.00059	benign	tolerated	benign	5.03

Legend: LoF = Loss-of-function

Supplementary Figure 1: Regional association result of genome-wide significant locus for chromosome 1, region 1.

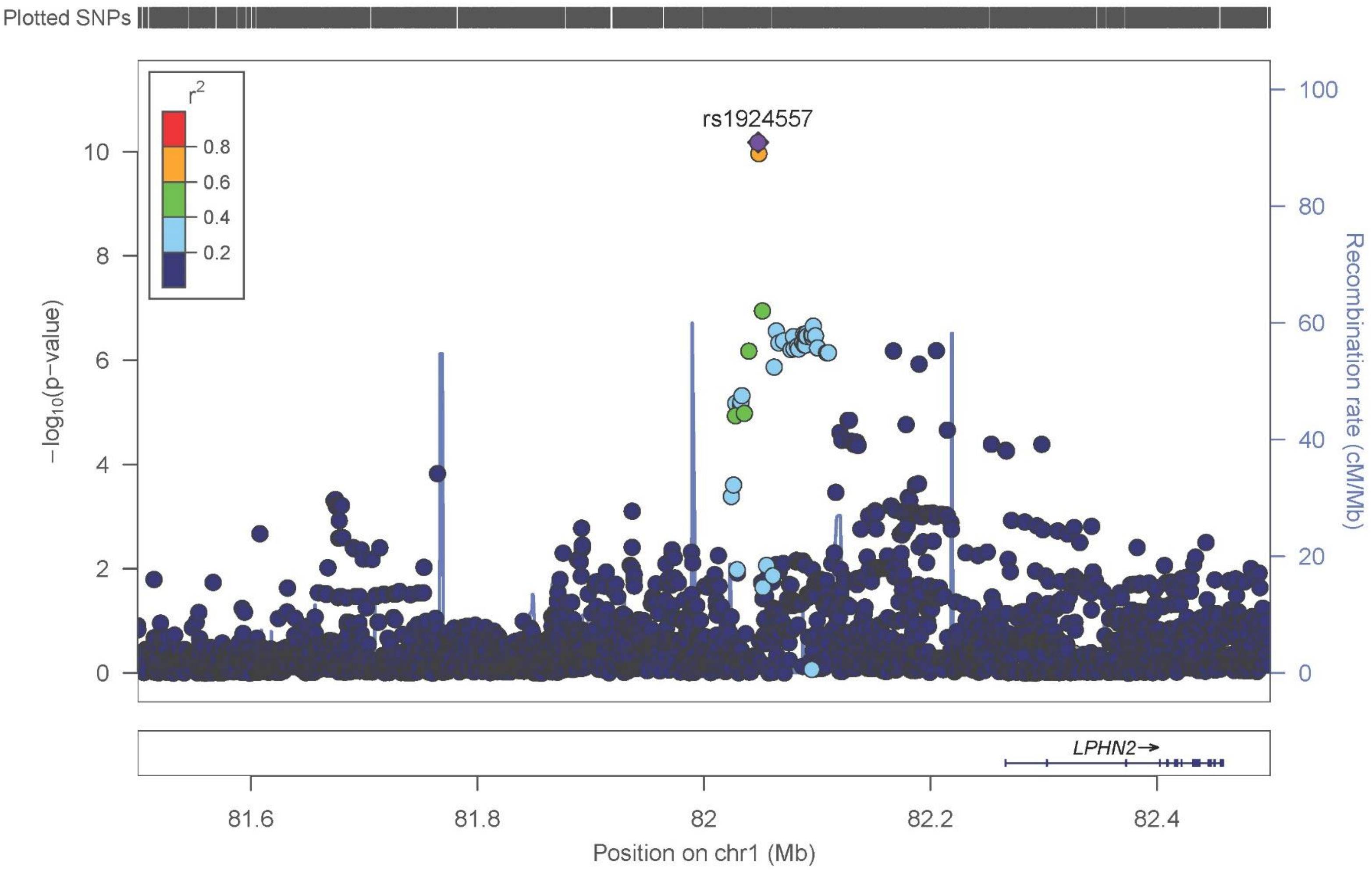

Every dot is a SNP in a 1 Megabase (Mb) window. *X*-axes represent chromosome position of the SNP and the relative genes position in the locus. *Y*-axes represent $-\log_{10}$(*P*-value) of each SNP. Blue peaks indicate the Recombination rate (cM/Mb). Colours of SNP represent the r^2 LD-block value. The most significant SNP is labelled in purple.

Supplementary Figure 2: Regional association result of genome-wide significant locus for chromosome 1, region 2.

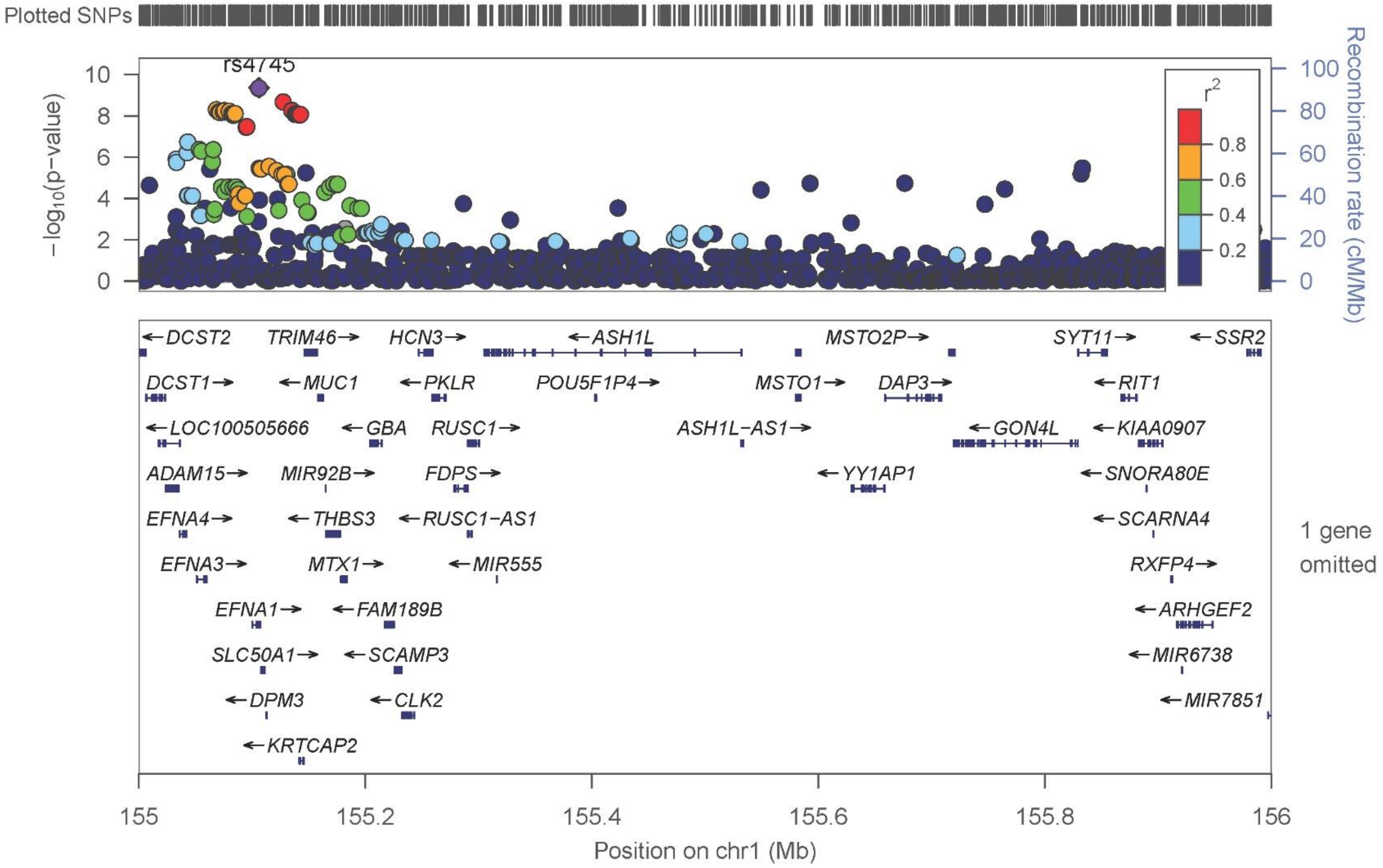

Every dot is a SNP in a 1 Megabase (Mb) window. *X*-axes represent chromosome position of the SNP and the relative genes position in the locus. *Y*-axes represent $-\log_{10}$(*P*-value) of each SNP. Blue peaks indicate the Recombination rate (cM/Mb). Colours of SNP represent the r^2 LD-block value. The most significant SNP is labelled in purple.

Supplementary Figure 3: Regional association result of genome-wide significant locus for chromosome 3.

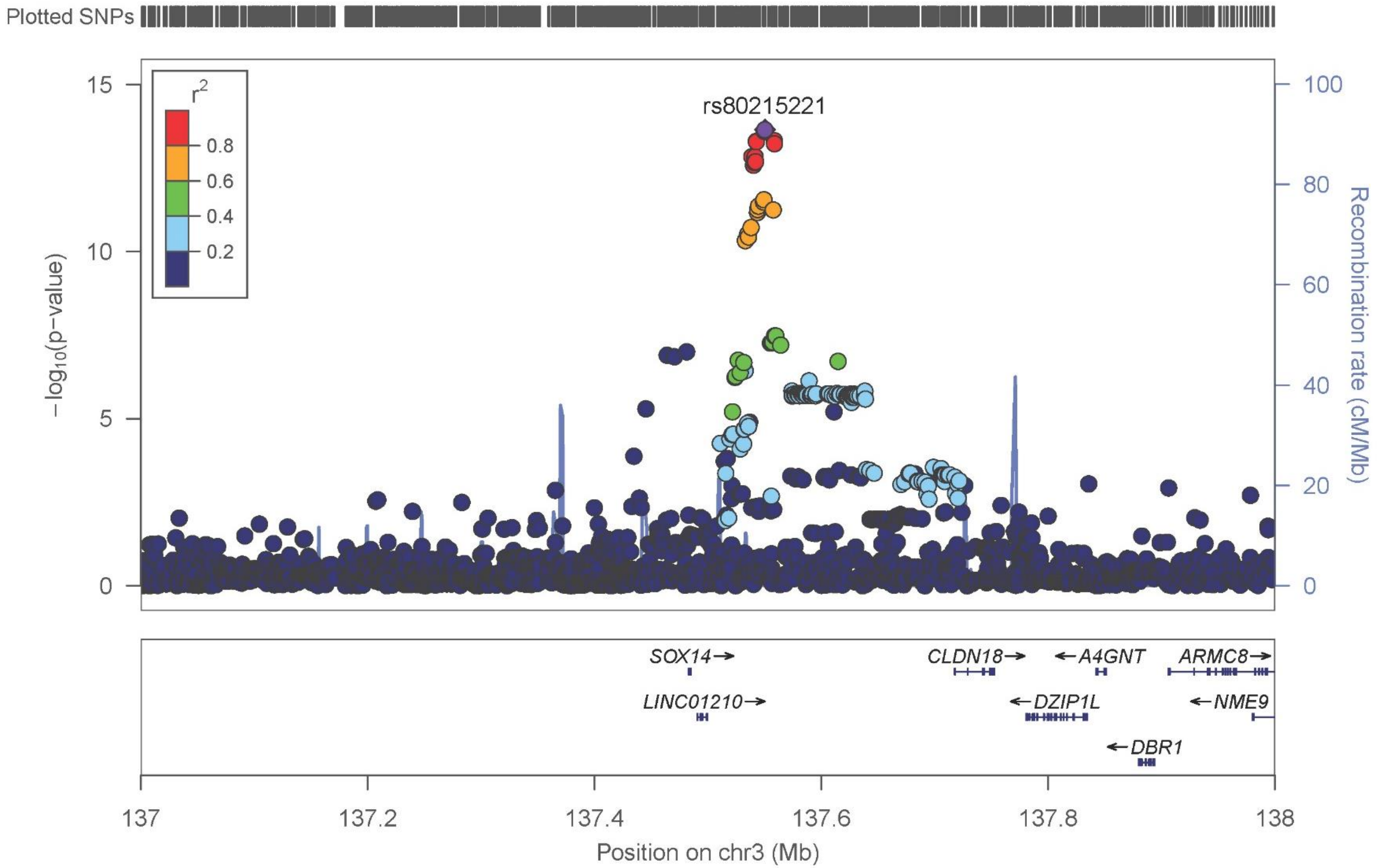

Every dot is a SNP in a 1 Megabase (Mb) window. *X*-axes represent chromosome position of the SNP and the relative genes position in the locus. *Y*-axes represent –log_{10}(*P*-value) of each SNP. Blue peaks indicate the Recombination rate (cM/Mb). Colours of SNP represent the r^2 LD-block value. The most significant SNP is labelled in purple.

Supplementary Figure 4: Regional association result of genome-wide significant locus for chromosome 5.

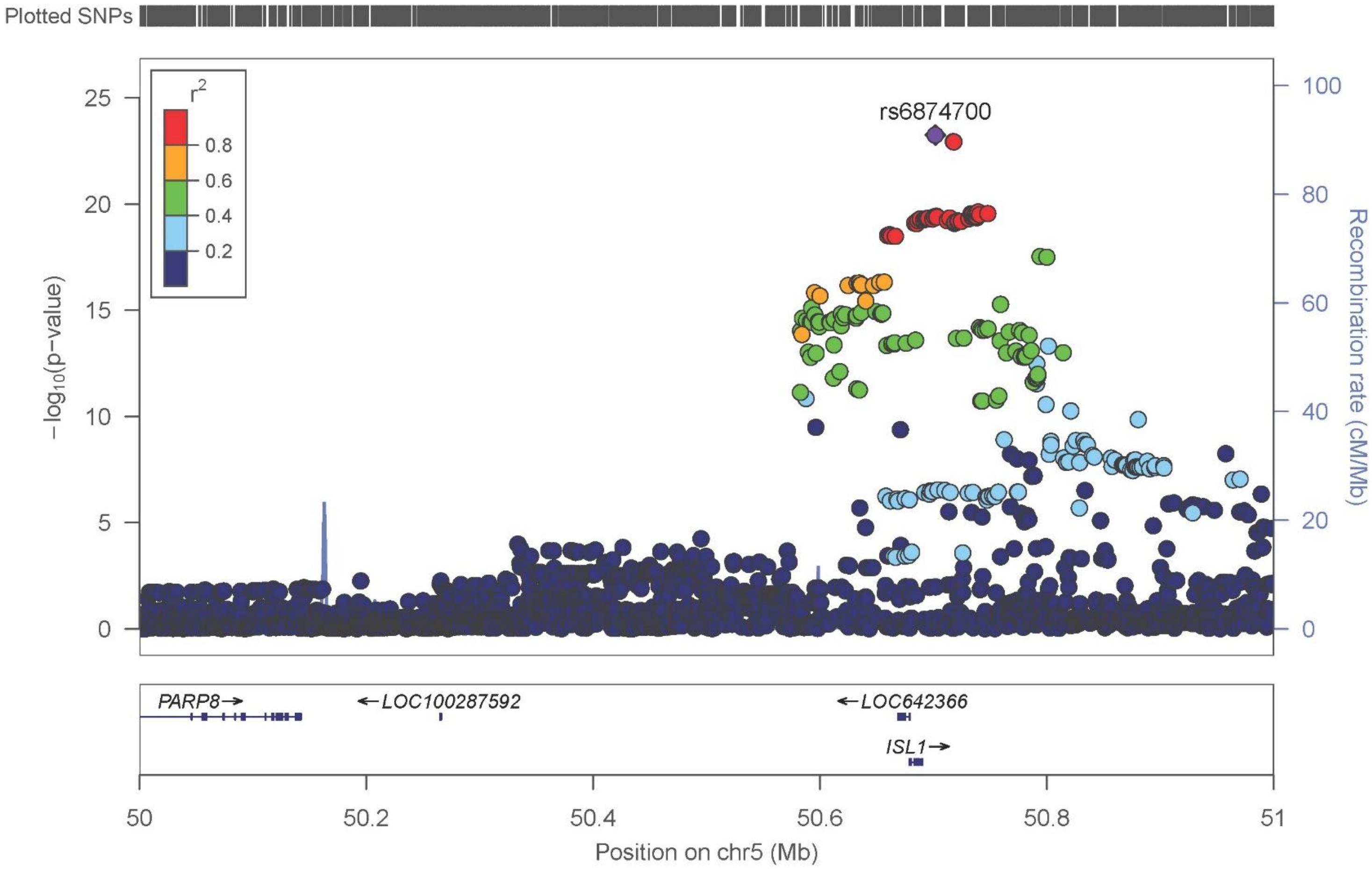

Every dot is a SNP in a 1 Megabase (Mb) window. *X*-axes represent chromosome position of the SNP and the relative genes position in the locus. *Y*-axes represent $-\log_{10}(P\text{-value})$ of each SNP. Blue peaks indicate the Recombination rate (cM/Mb). Colours of SNP represent the r^2 LD-block value. The most significant SNP is labelled in purple.

Supplementary Figure 5: Regional association result of genome-wide significant locus for chromosome 11

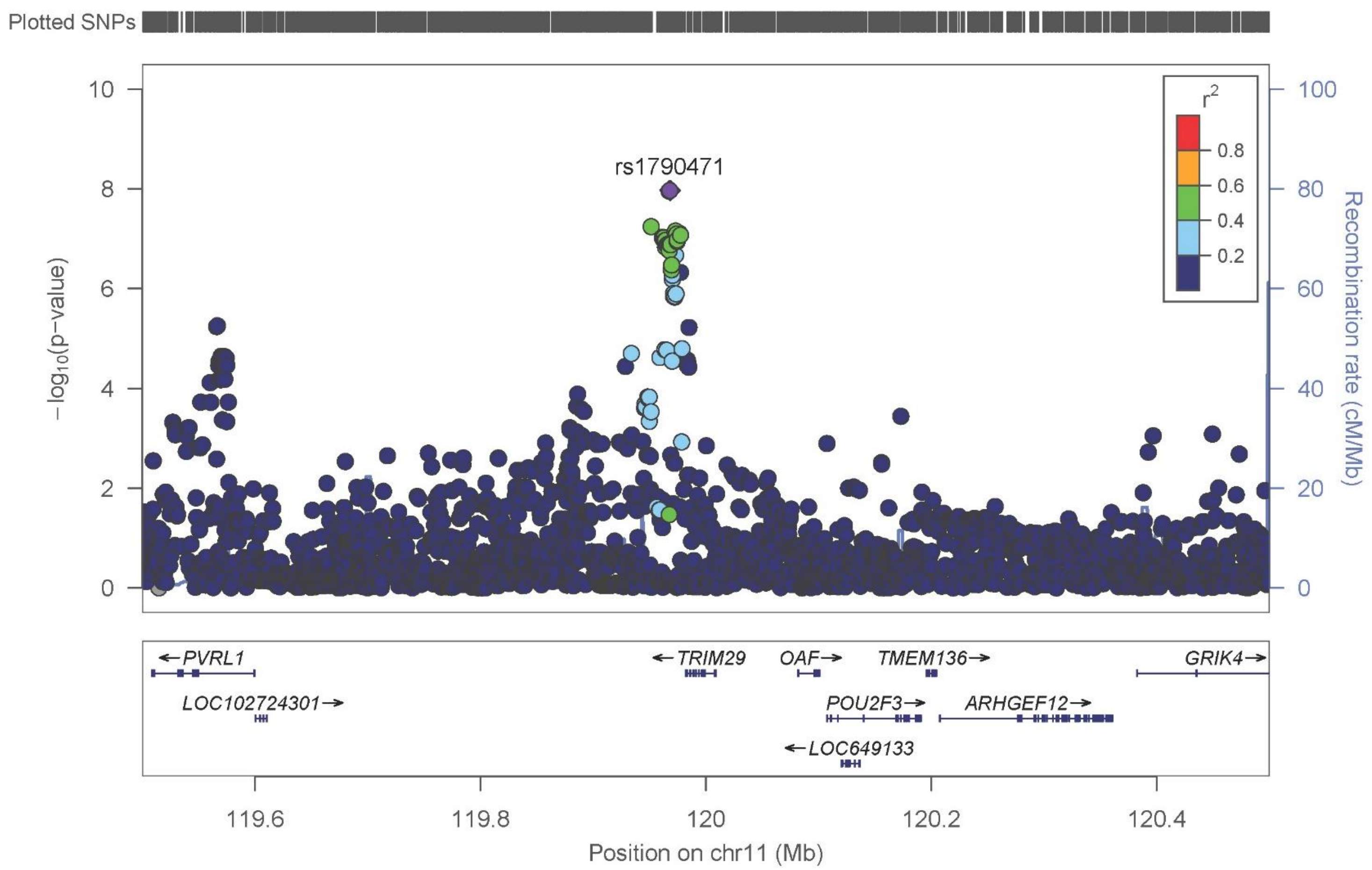

Every dot is a SNP in a 1 Megabase (Mb) window. *X*-axes represent chromosome position of the SNP and the relative genes position in the locus. *Y*-axes represent $-\log_{10}$(*P*-value) of each SNP. Blue peaks indicate the Recombination rate (cM/Mb). Colours of SNP represent the r^2 LD-block value. The most significant SNP is labelled in purple.

Supplementary Figure 6: Regional association result of genome-wide significant locus for chromosome 12

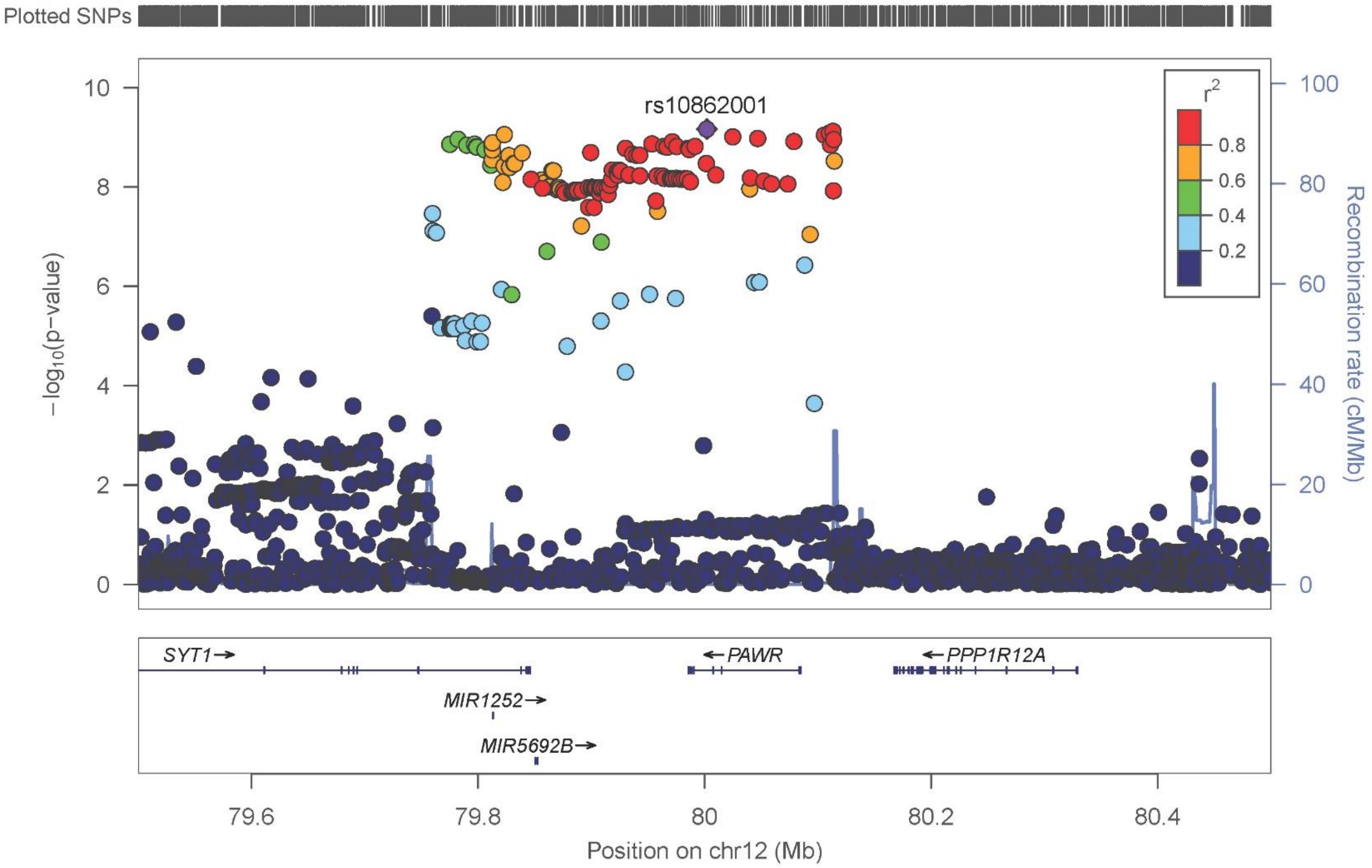

Every dot is a SNP in a 1 Megabase (Mb) window. *X*-axes represent chromosome position of the SNP and the relative genes position in the locus. *Y*-axes represent –$\log_{10}$(*P*-value) of each SNP. Blue peaks indicate the Recombination rate (cM/Mb). Colours of SNP represent the r^2 LD-block value. The most significant SNP is labelled in purple.

Supplementary Figure 7: Regional association result of genome-wide significant locus for chromosome 17

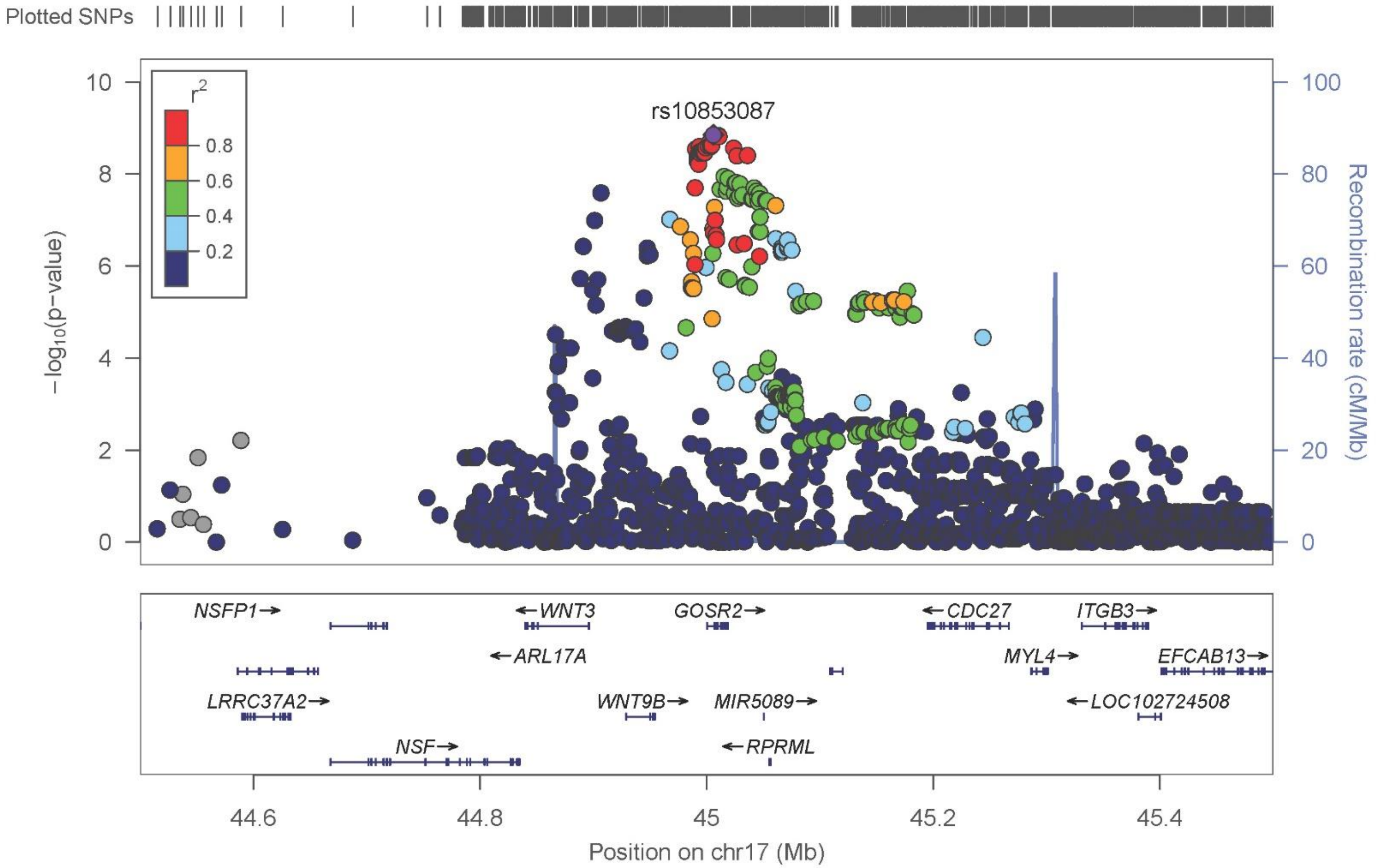

Every dot is a SNP in a 1 Megabase (Mb) window. *X*-axes represent chromosome position of the SNP and the relative genes position in the locus. *Y*-axes represent $-\log_{10}$(*P*-value) of each SNP. Blue peaks indicate the Recombination rate (cM/Mb). Colours of SNP represent the r^2 LD-block value. The most significant SNP is labelled in purple.

Supplementary Figure 8: Regional association result of genome-wide significant locus for chromosome 20

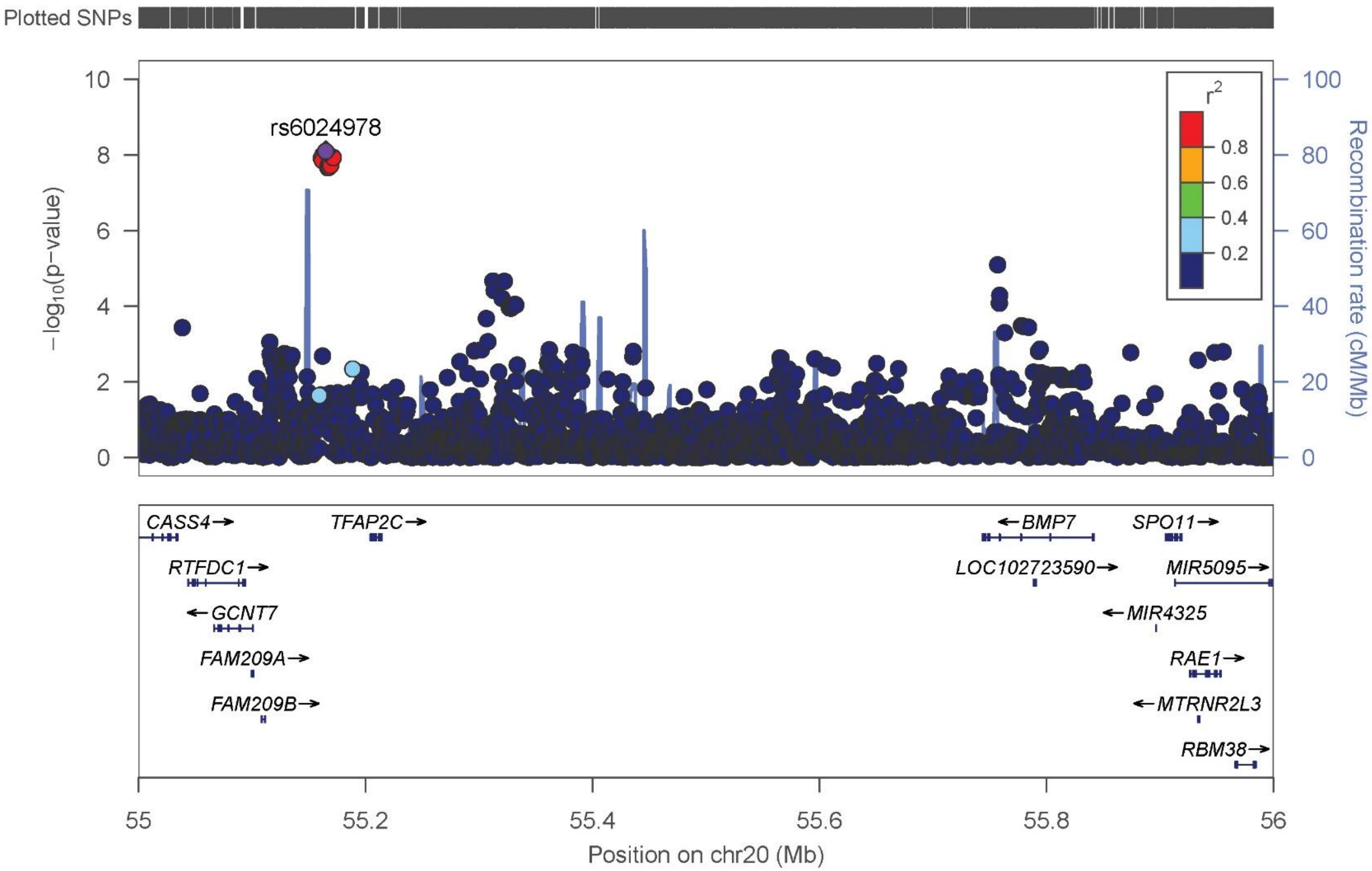

Every dot is a SNP in a 1 Megabase (Mb) window. *X*-axes represent chromosome position of the SNP and the relative genes position in the locus. *Y*-axes represent –$\log_{10}$(*P*-value) of each SNP. Blue peaks indicate the Recombination rate (cM/Mb). Colours of SNP represent the r^2 LD-block value. The most significant SNP is labelled in purple.

Supplementary Figure 9: Principal component analysis of Central Europe cohort.

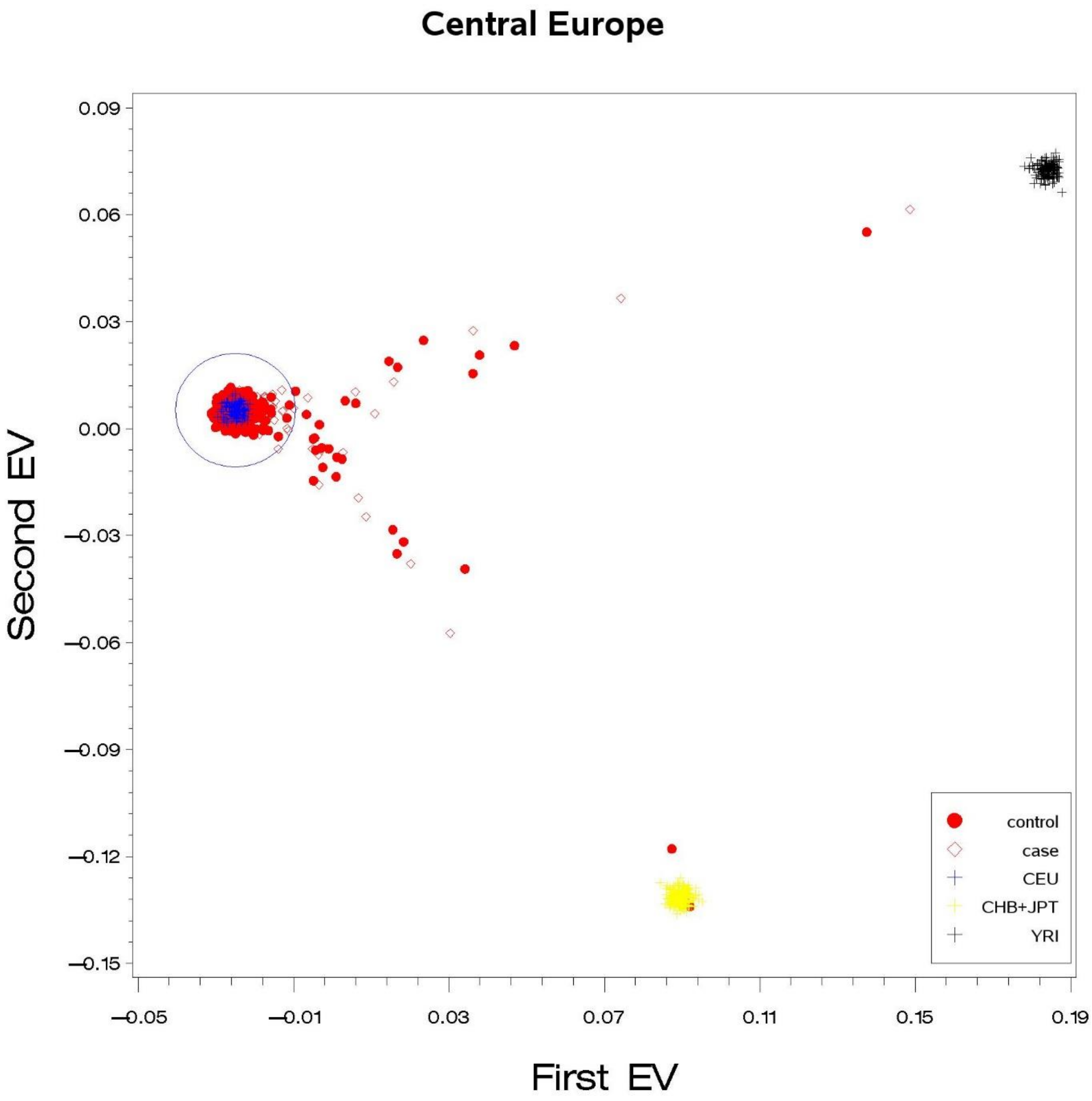

Only individuals within the red ellipses defined by condition (1) were used for the statistical analysis, individuals outside the red ellipsis were discarded. (CEU) Utah Residents from North and West Europe; (CHB) Han Chinese in Beijing, China; (JPT) Japanese in Tokyo, Japan; (YRI) Yoruba in Ibadan, Nigeria.

Supplementary Figure 10: Principal component analysis of Spain cohort.

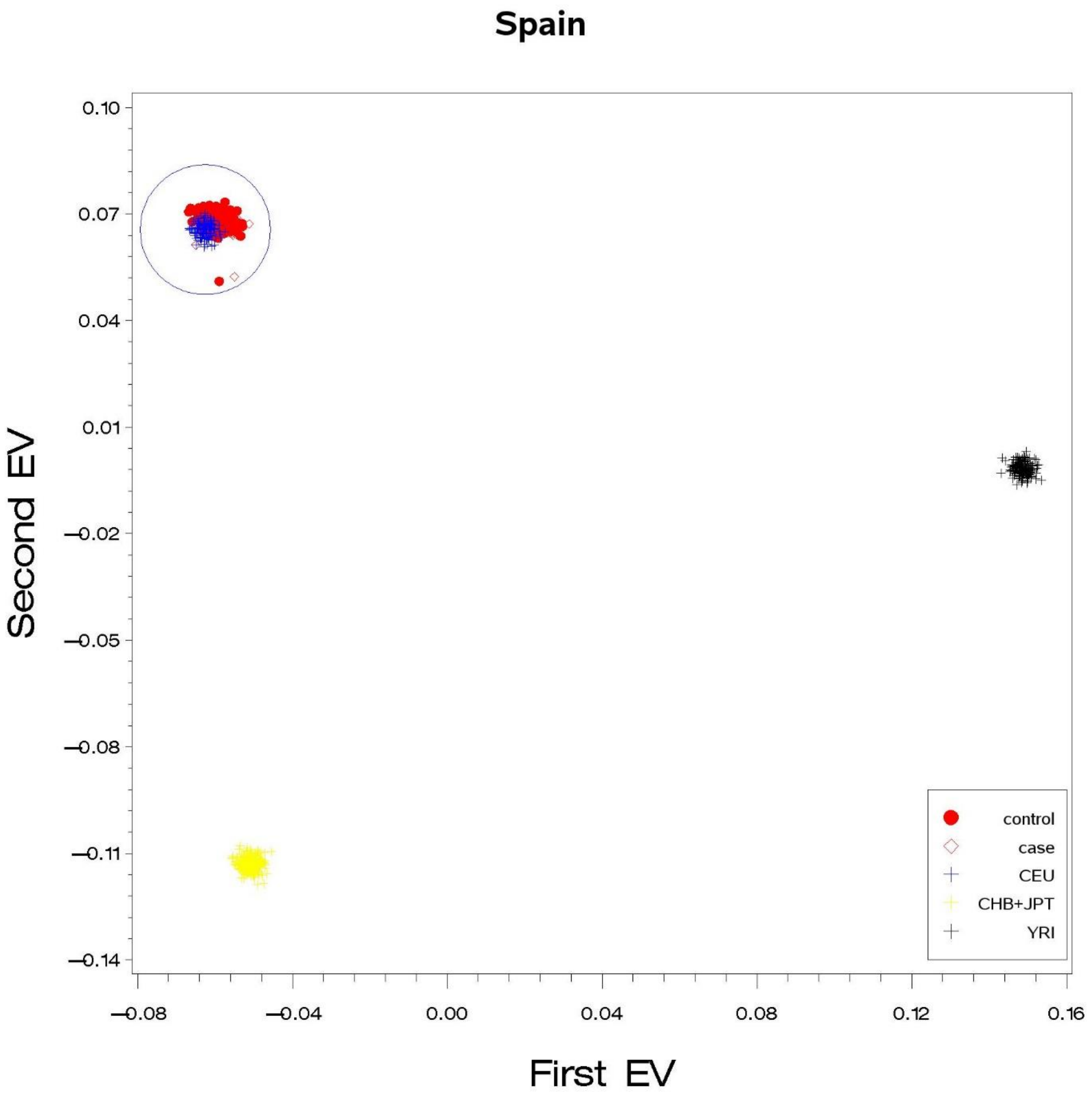

Only individuals within the red ellipses defined by condition (1) were used for the statistical analysis, individuals outside the red ellipsis were discarded. (CEU) Utah Residents from North and West Europe; (CHB) Han Chinese in Beijing, China; (JPT) Japanese in Tokyo, Japan; (YRI) Yoruba in Ibadan, Nigeria.

Supplementary Figure 11: Principal component analysis of Italy cohort.

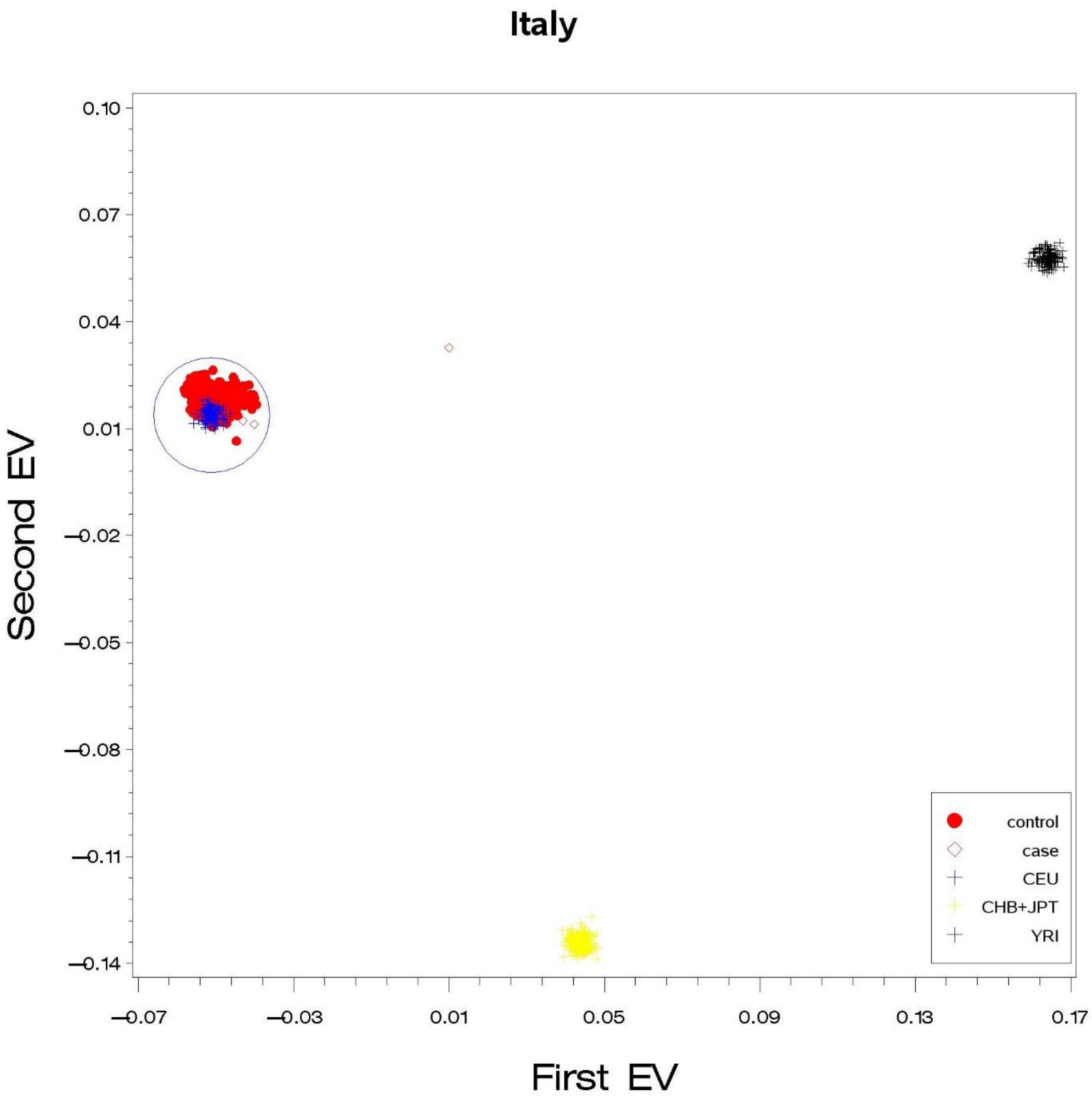

Only individuals within the red ellipses defined by condition (1) were used for the statistical analysis, individuals outside the red ellipsis were discarded. (CEU) Utah Residents from North and West Europe; (CHB) Han Chinese in Beijing, China; (JPT) Japanese in Tokyo, Japan; (YRI) Yoruba in Ibadan, Nigeria.

Supplementary Figure 12: Principal component analysis of UK cohort

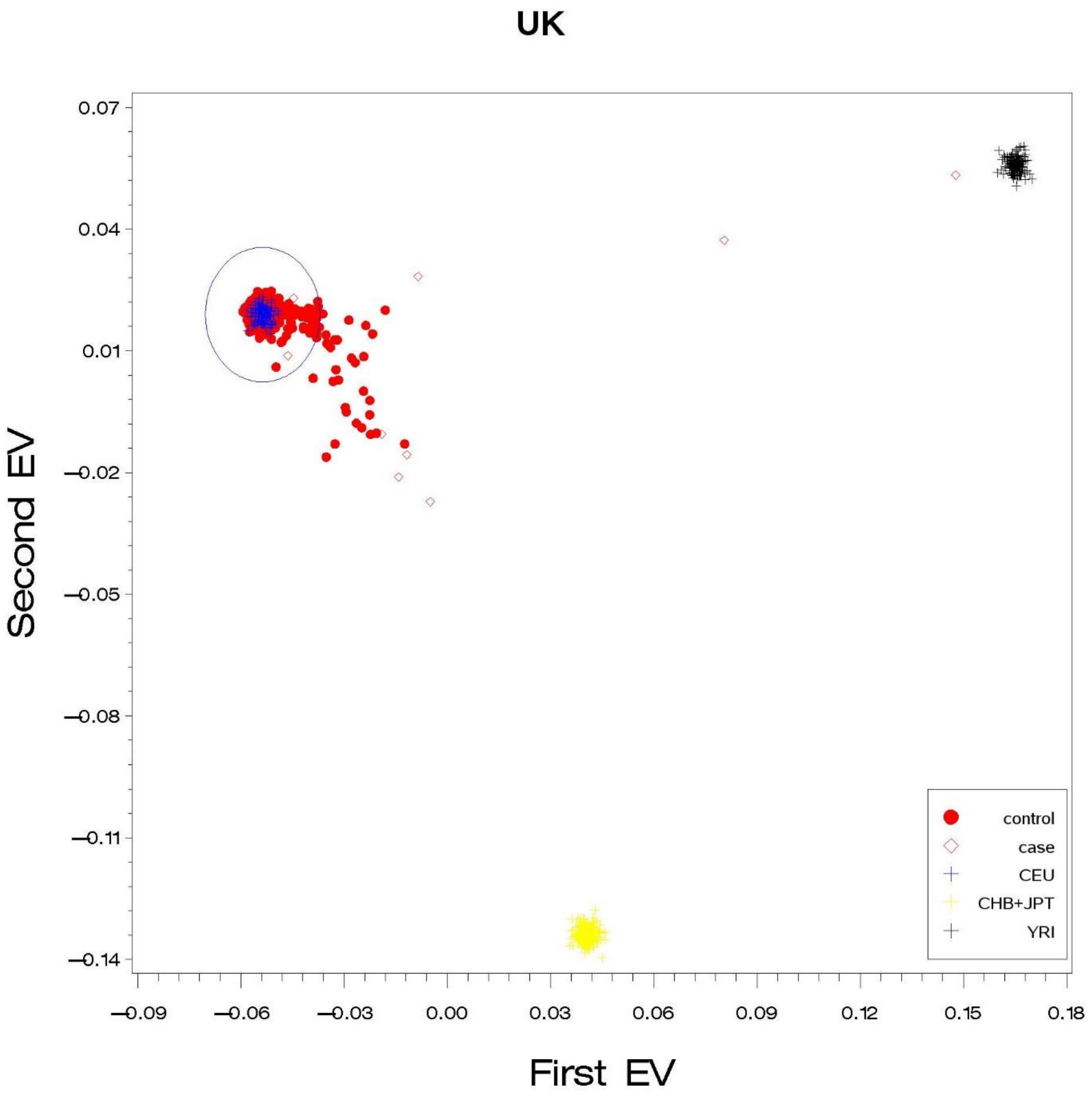

Only individuals within the red ellipses defined by condition (1) were used for the statistical analysis, individuals outside the red ellipsis were discarded. (CEU) Utah Residents from North and West Europe; (CHB) Han Chinese in Beijing, China; (JPT) Japanese in Tokyo, Japan; (YRI) Yoruba in Ibadan, Nigeria.

Supplementary Figure 13: Principal component analysis of Sweden cohort

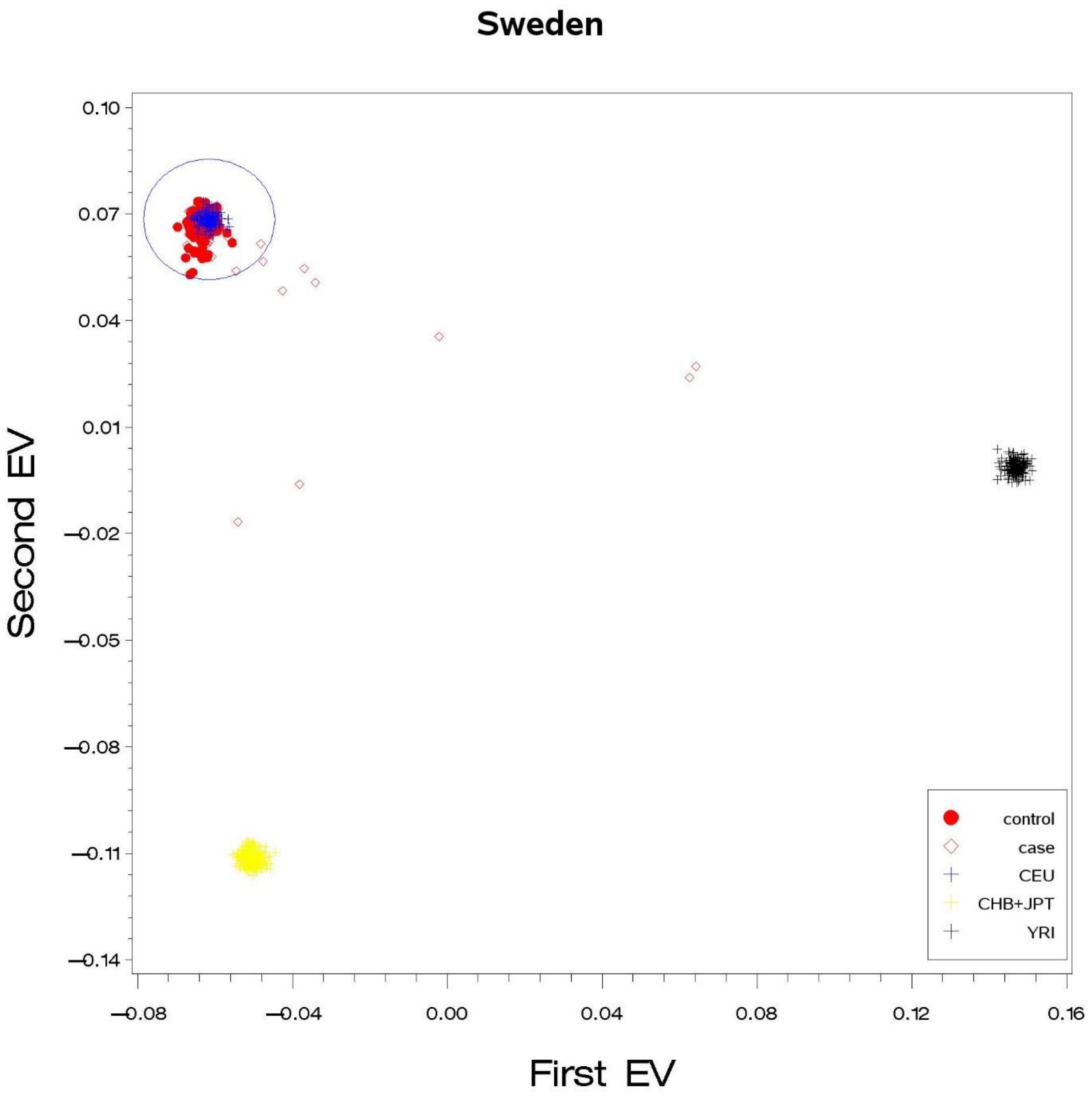

Only individuals within the red ellipses defined by condition (1) were used for the statistical analysis, individuals outside the red ellipsis were discarded. (CEU) Utah Residents from North and West Europe; (CHB) Han Chinese in Beijing, China; (JPT) Japanese in Tokyo, Japan; (YRI) Yoruba in Ibadan, Nigeria.

Supplementary Figure 14: Principal component analysis of GWAS 1 strata.

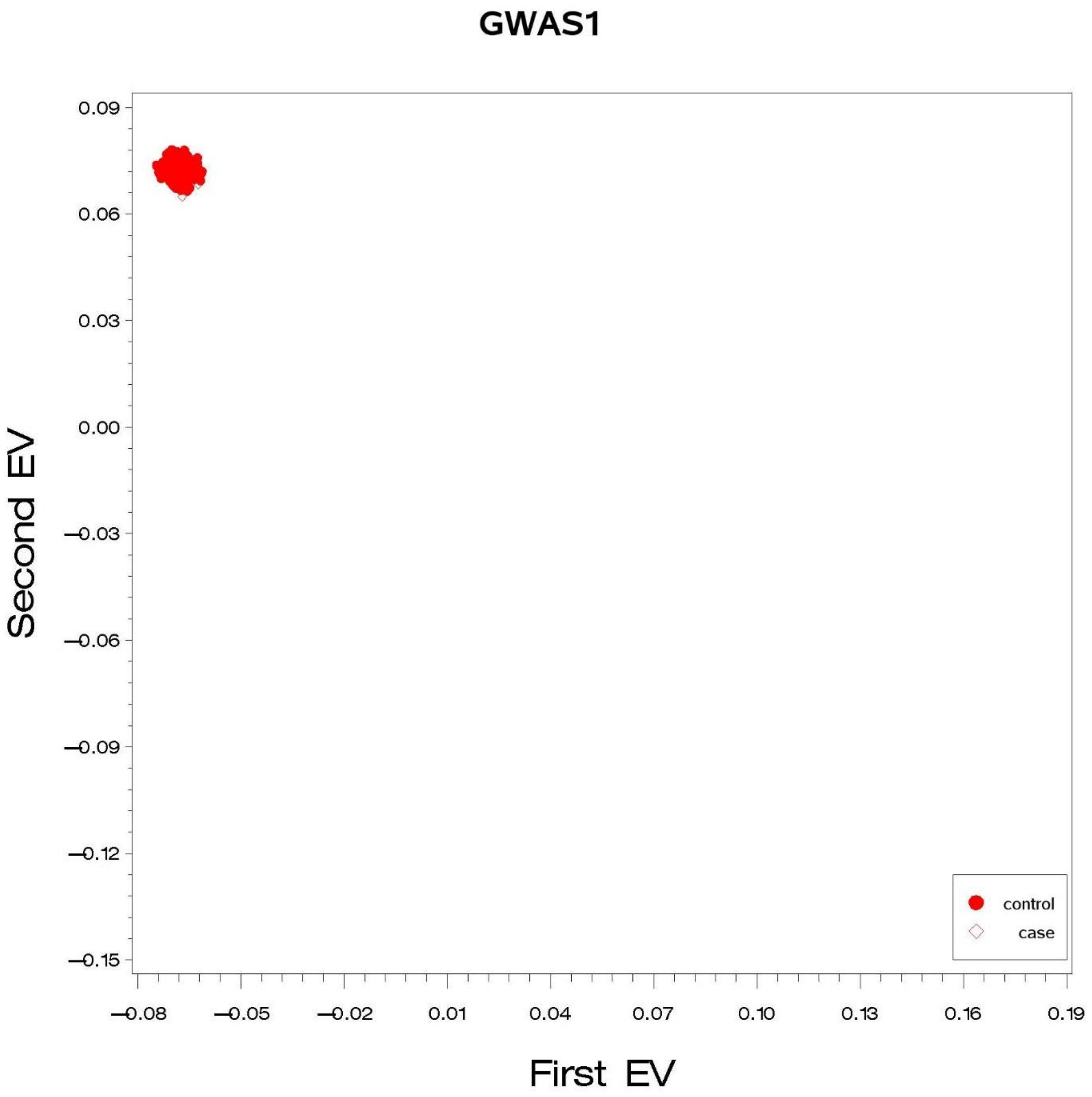

Only individuals within the red ellipses defined by condition (1) were used for the statistical analysis, individuals outside the red ellipsis were discarded. (CEU) Utah Residents from North and West Europe; (CHB) Han Chinese in Beijing, China; (JPT) Japanese in Tokyo, Japan; (YRI) Yoruba in Ibadan, Nigeria.

Supplementary Figure 15: Principal component analysis of GWAS 2 strata.

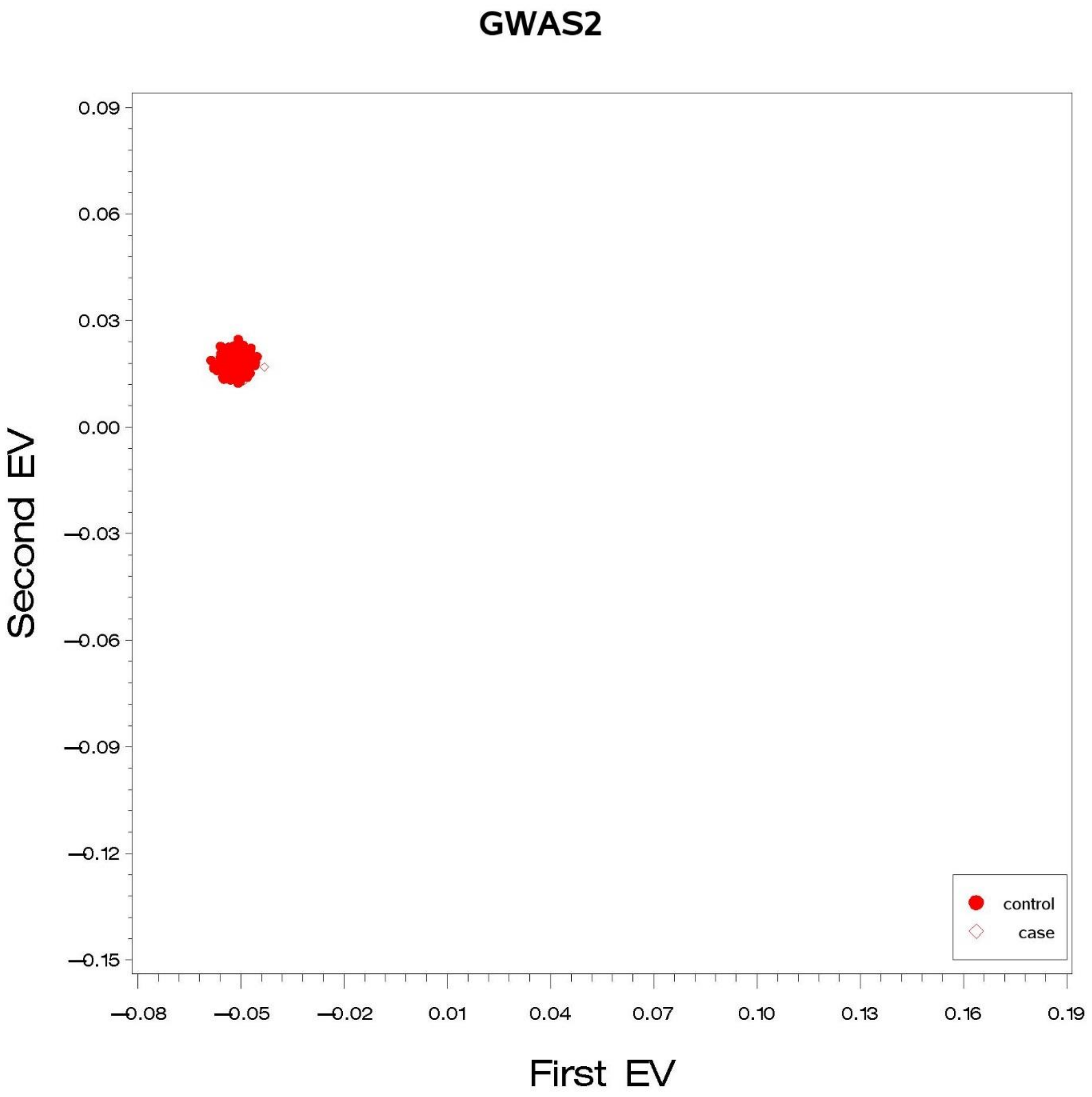

Only individuals within the red ellipses defined by condition (1) were used for the statistical analysis, individuals outside the red ellipsis were discarded. (CEU) Utah Residents from North and West Europe; (CHB) Han Chinese in Beijing, China; (JPT) Japanese in Tokyo, Japan; (YRI) Yoruba in Ibadan, Nigeria.

Supplementary Fig. 16: Meta-analysis Q-Q plot

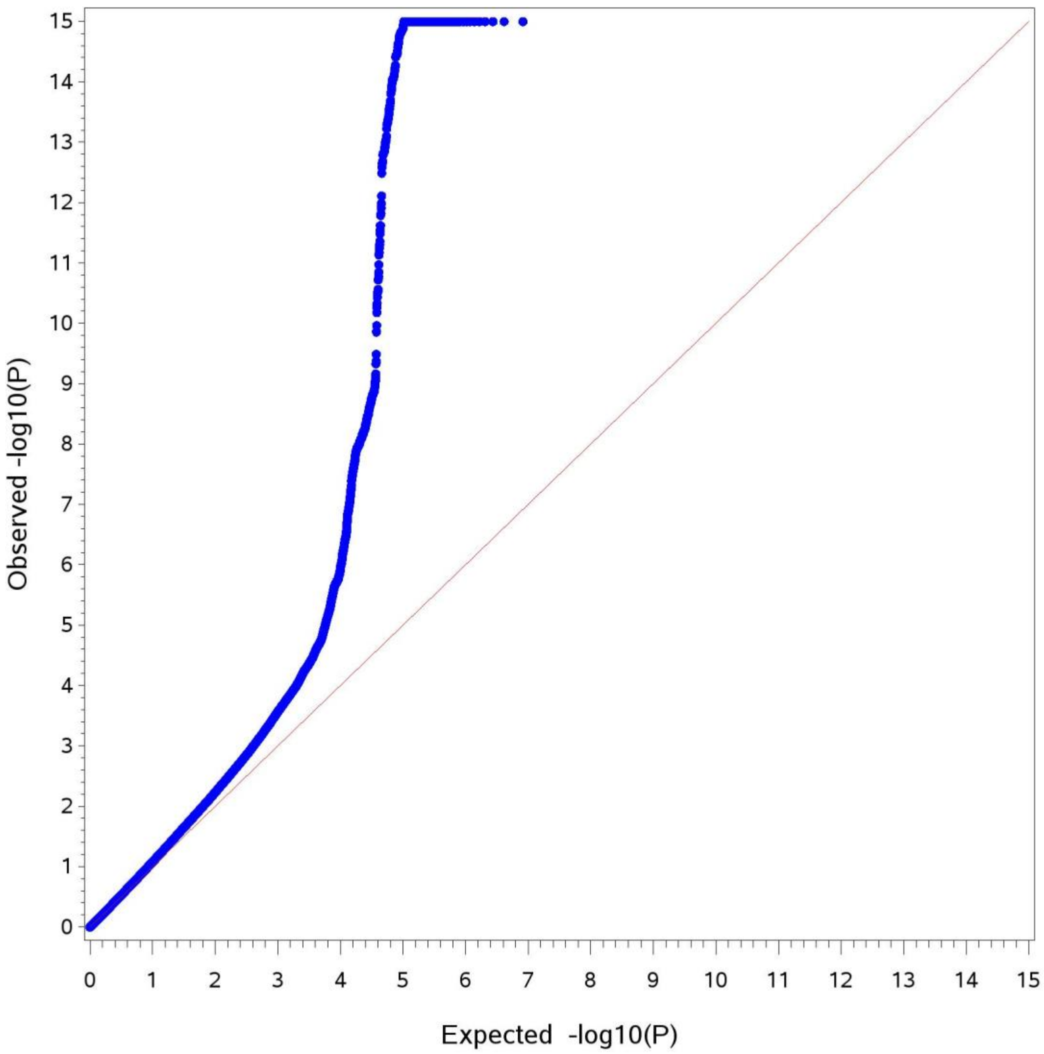

This Figure shows the Q-Q plots for the two-sided *P*-values obtained from the GWAS meta-analysis of BEEC. The X axis shows the expected distribution of -log10(*P*-values) under the null hypothesis of no association. The Y axis shows the distribution of the observed -log10(*P*-values) in the meta-analysis. The red line indicates where Y=X.

Supplementary Fig. 17: Manhattan plot of the Genome-Wide Association Studies for the 628 CBE patients and 7,352 ethnically matched controls.

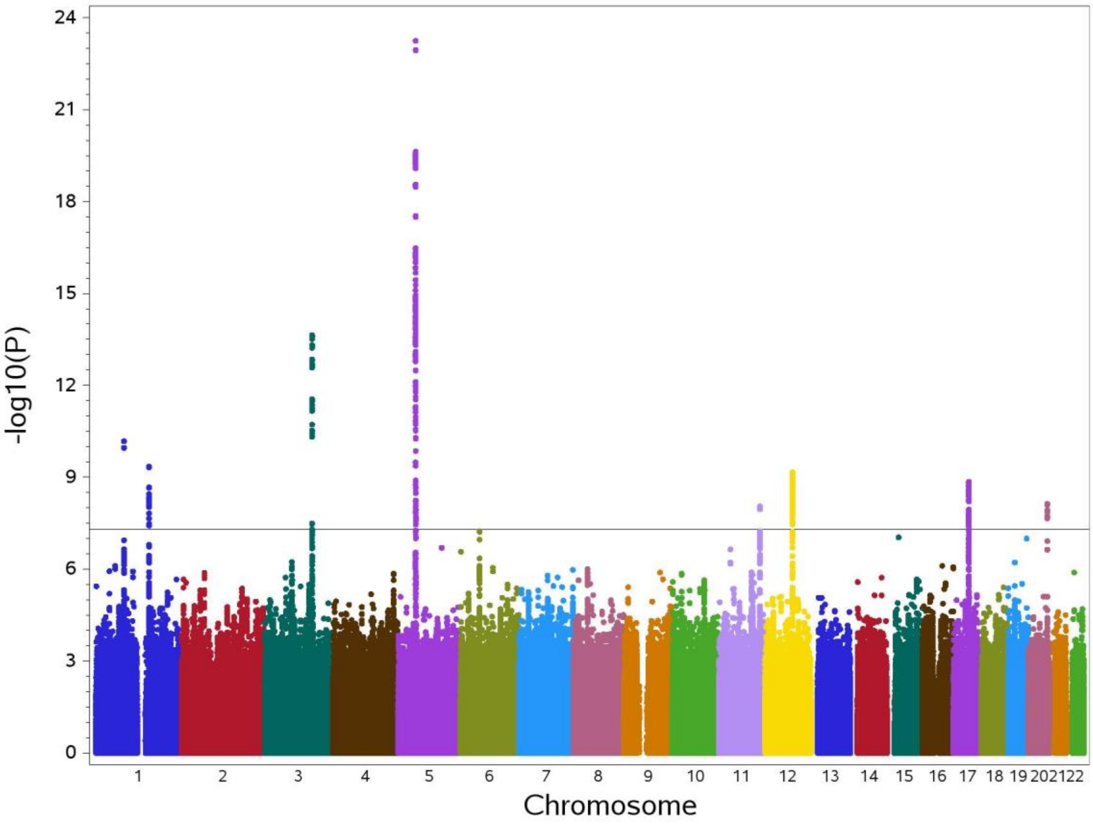

X-axis shows the chromosomes with each dot representing a SNP. *Y*-axis shows -$\log_{10}$(*P*-value) of the association of each SNP in the CBE cohort. Continue black horizontal line indicate the threshold of the genome-wide significance at *P*-value of $5*10^{-8}$.

Supplementary Fig. 18: Anatomical structures and details of mouse embryos dissected tissues for RNA-seq.

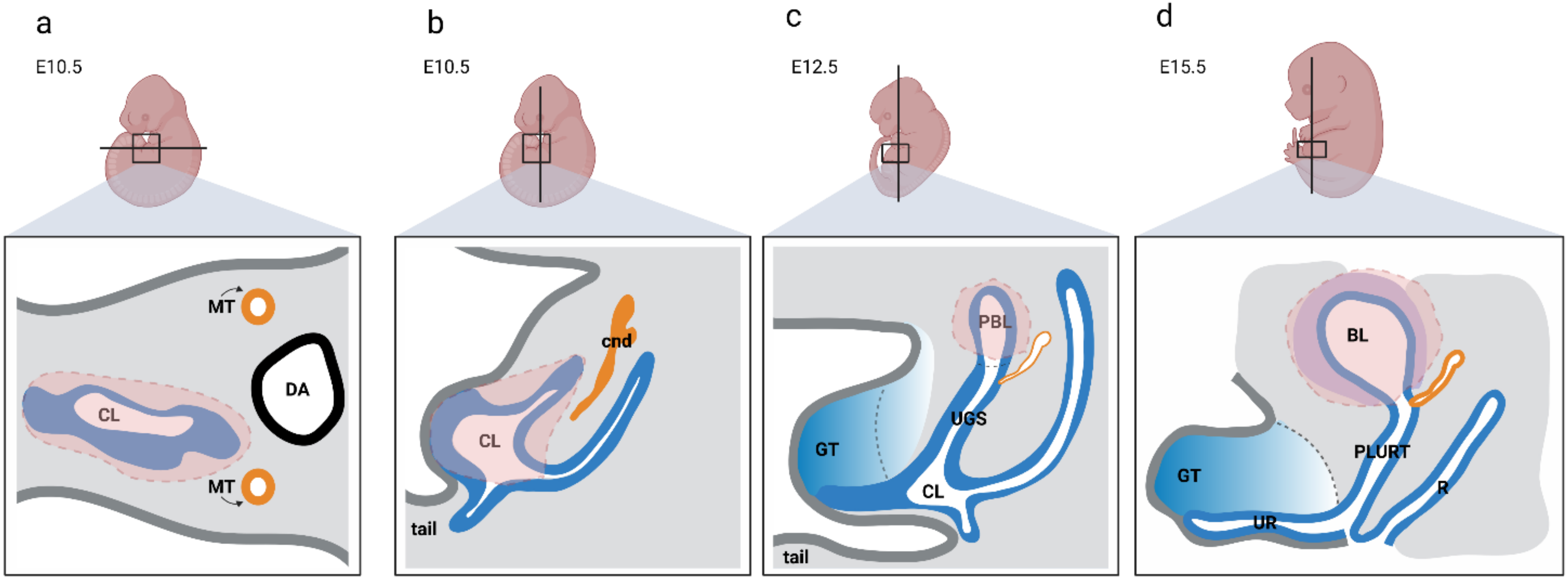

a) Embryonic day E10.5, transversal section. **b**) Embryonic day E10.5, sagittal section. **c**) Embryonic day E12.5, sagittal section. **d**) Embryonic day E15.5, sagittal section. Nomenclature: cloaca (CL); dorsal aorta (DA); mesonephric tubules (MT); common nephric duct (cnd); genital tubercle (GT); urogenital sinus (UGS); primitive bladder (PBL); urethra (UR); pelvic urethra (PLURT); rectum (R); bladder (BL).

Supplementary Fig. 19: Timelines of mouse embryonic and human embryonic and fetal bladder sampling for RNA-seq used in this study.

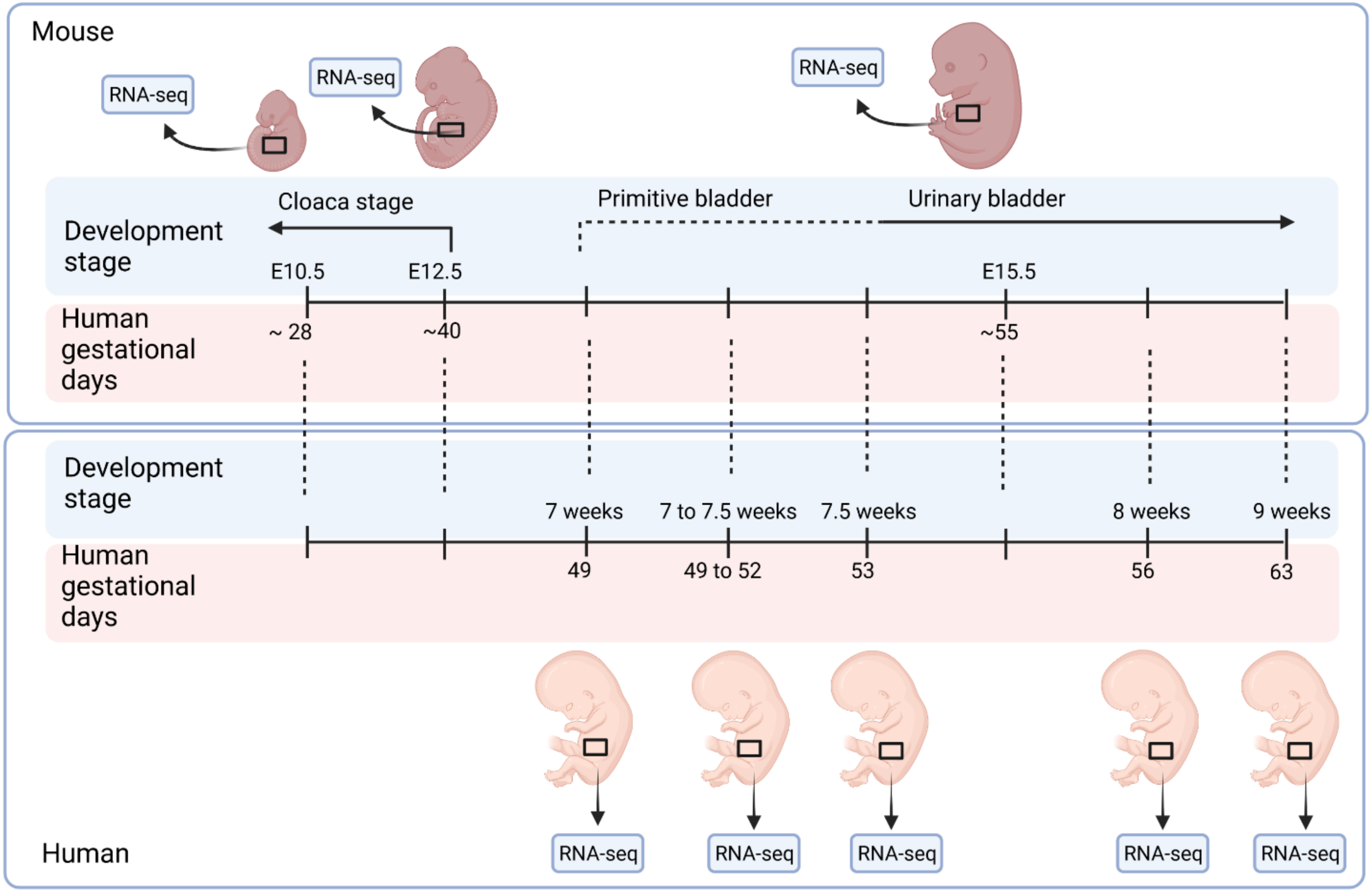

Supplementary Fig. 20: Regional association plots for conditional logistic regression in genome-wide significant locus in chromosome 1, region 1.

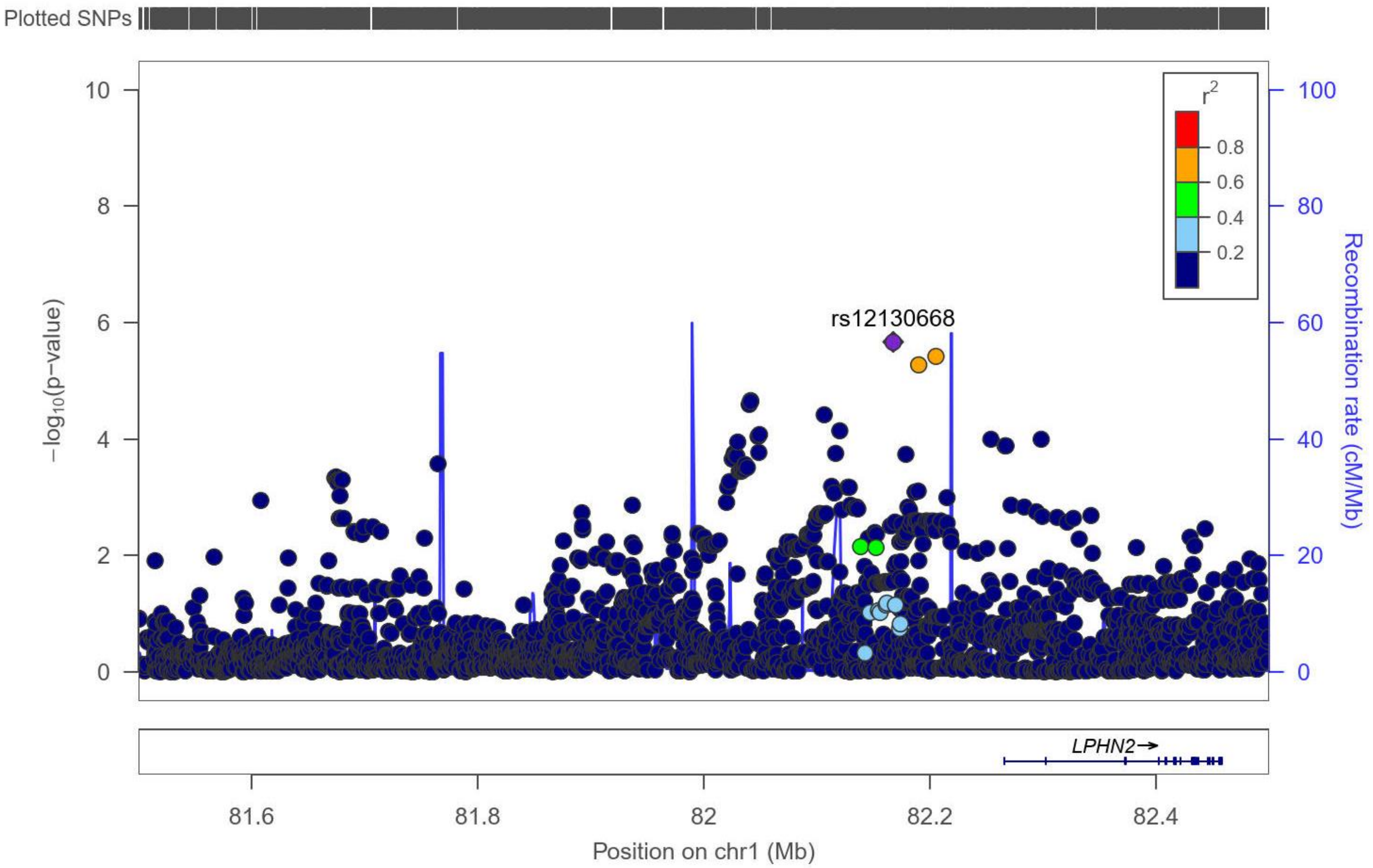

Every dot is a SNP in a 1 Megabase (Mb) window. *X*-axes represent chromosome position of the SNP and the relative genes position in the locus. *Y*-axes represent $-\log_{10}$(*P*-value) of each SNP. Blue peaks indicate the Recombination rate (cM/Mb). The most significant SNP is labelled in purple.

Supplementary Fig. 21: Regional association plots for conditional logistic regression in genome-wide significant locus in chromosome 1, region 2.

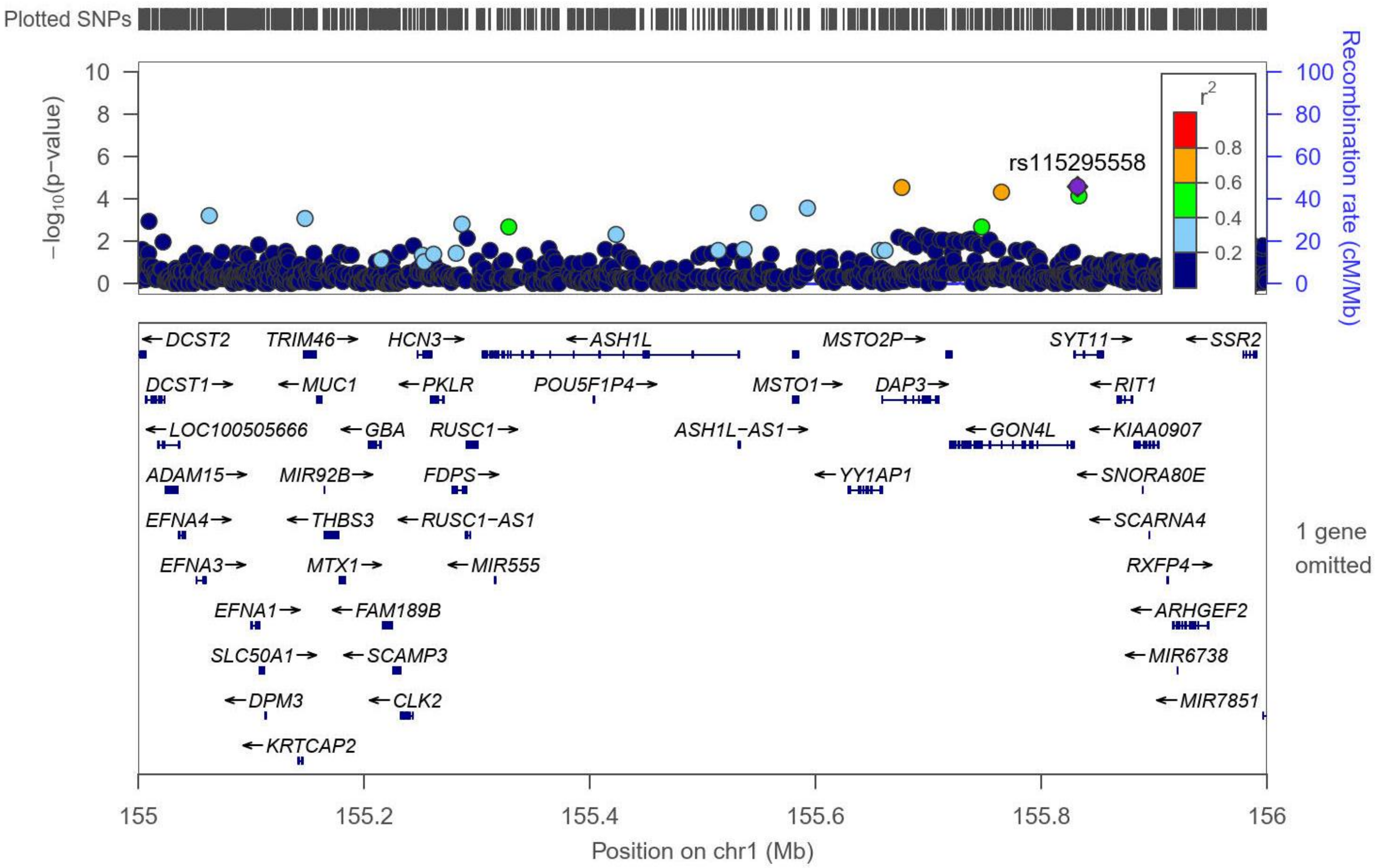

Every dot is a SNP in a 1 Megabase (Mb) window. *X*-axes represent chromosome position of the SNP and the relative genes position in the locus. *Y*-axes represent –log10(*P*-value) of each SNP. Blue peaks indicate the Recombination rate (cM/Mb). The most significant SNP is labelled in purple.

Supplementary Fig. 22: Regional association plots for conditional logistic regression in genome-wide significant locus in chromosome 3.

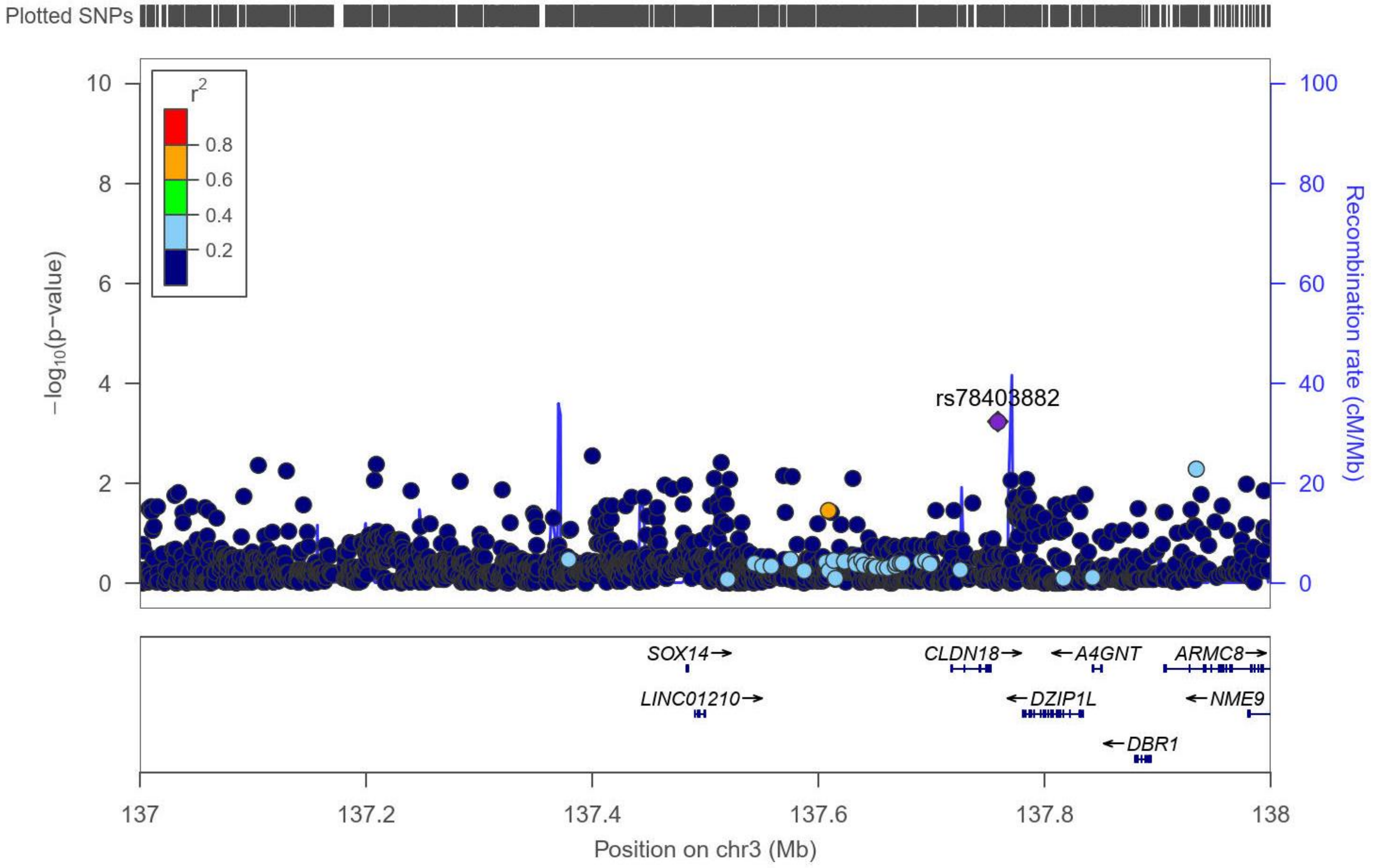

Every dot is a SNP in a 1 Megabase (Mb) window. *X*-axes represent chromosome position of the SNP and the relative genes position in the locus. *Y*-axes represent –$\log_{10}$(*P*-value) of each SNP. Blue peaks indicate the Recombination rate (cM/Mb). The most significant SNP is labelled in purple.

Supplementary Fig. 23: Regional association plots for conditional logistic regression in genome-wide significant locus in chromosome 5.

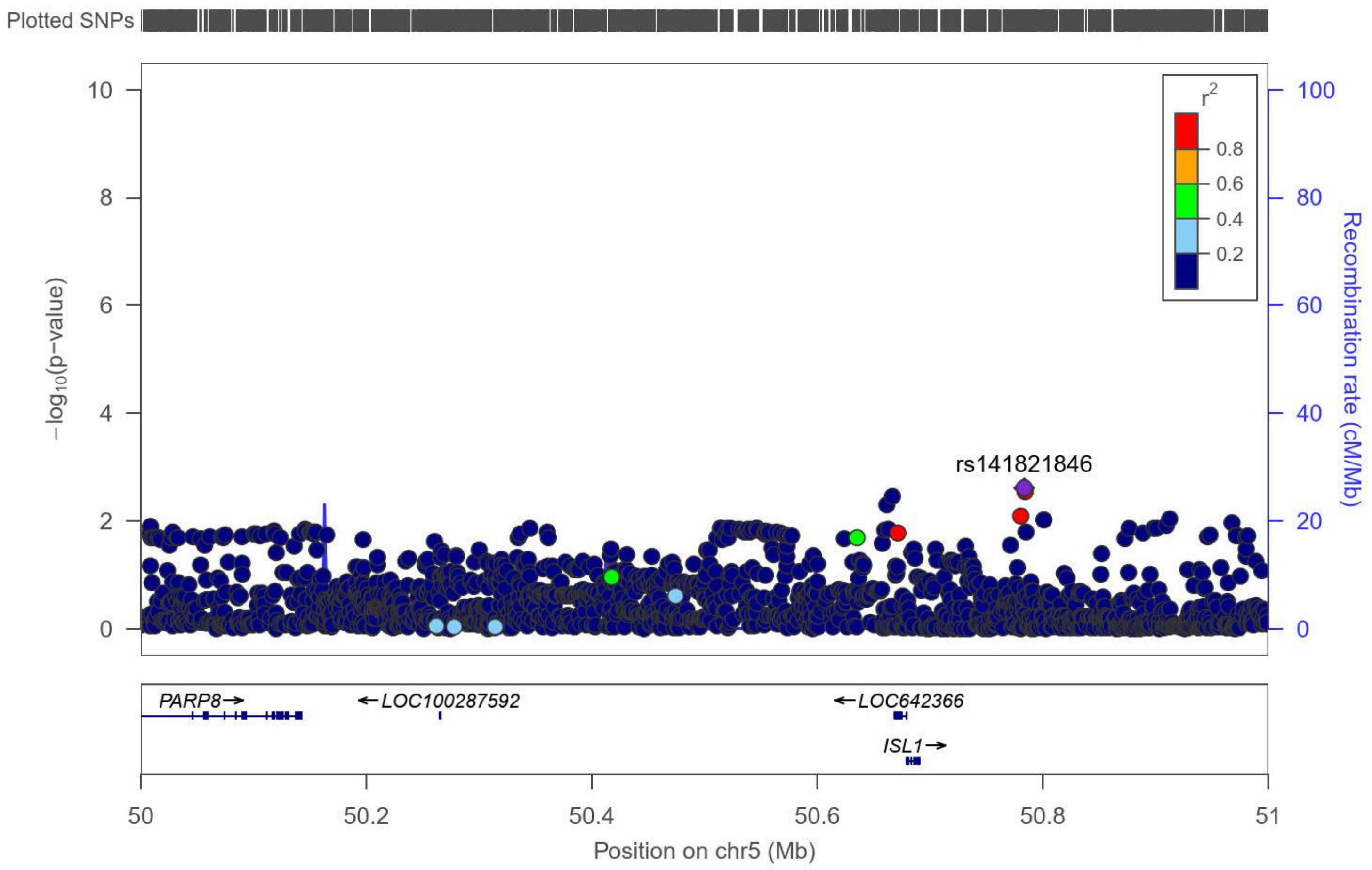

Every dot is a SNP in a 1 Megabase (Mb) window. *X*-axes represent chromosome position of the SNP and the relative genes position in the locus. *Y*-axes represent $-\log_{10}$(*P*-value) of each SNP. Blue peaks indicate the Recombination rate (cM/Mb). The most significant SNP is labelled in purple.

Supplementary Fig. 24: Regional association plots for conditional logistic regression in genome-wide significant locus in chromosome 11.

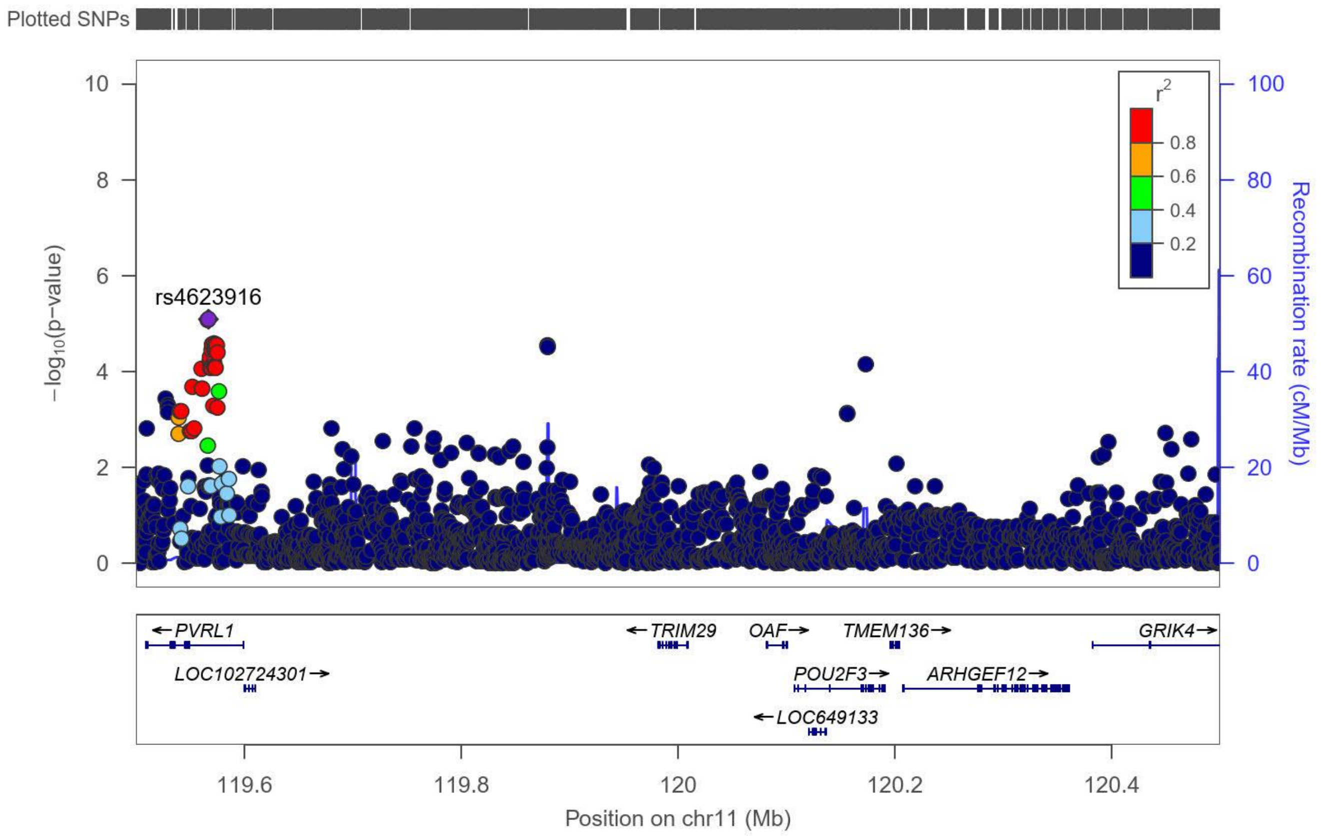

Every dot is a SNP in a 1 Megabase (Mb) window. *X*-axes represent chromosome position of the SNP and the relative genes position in the locus. *Y*-axes represent $-\log_{10}$(*P*-value) of each SNP. Blue peaks indicate the Recombination rate (cM/Mb). The most significant SNP is labelled in purple.

Supplementary Fig. 25: Regional association plots for conditional logistic regression in genome-wide significant locus in chromosome 12.

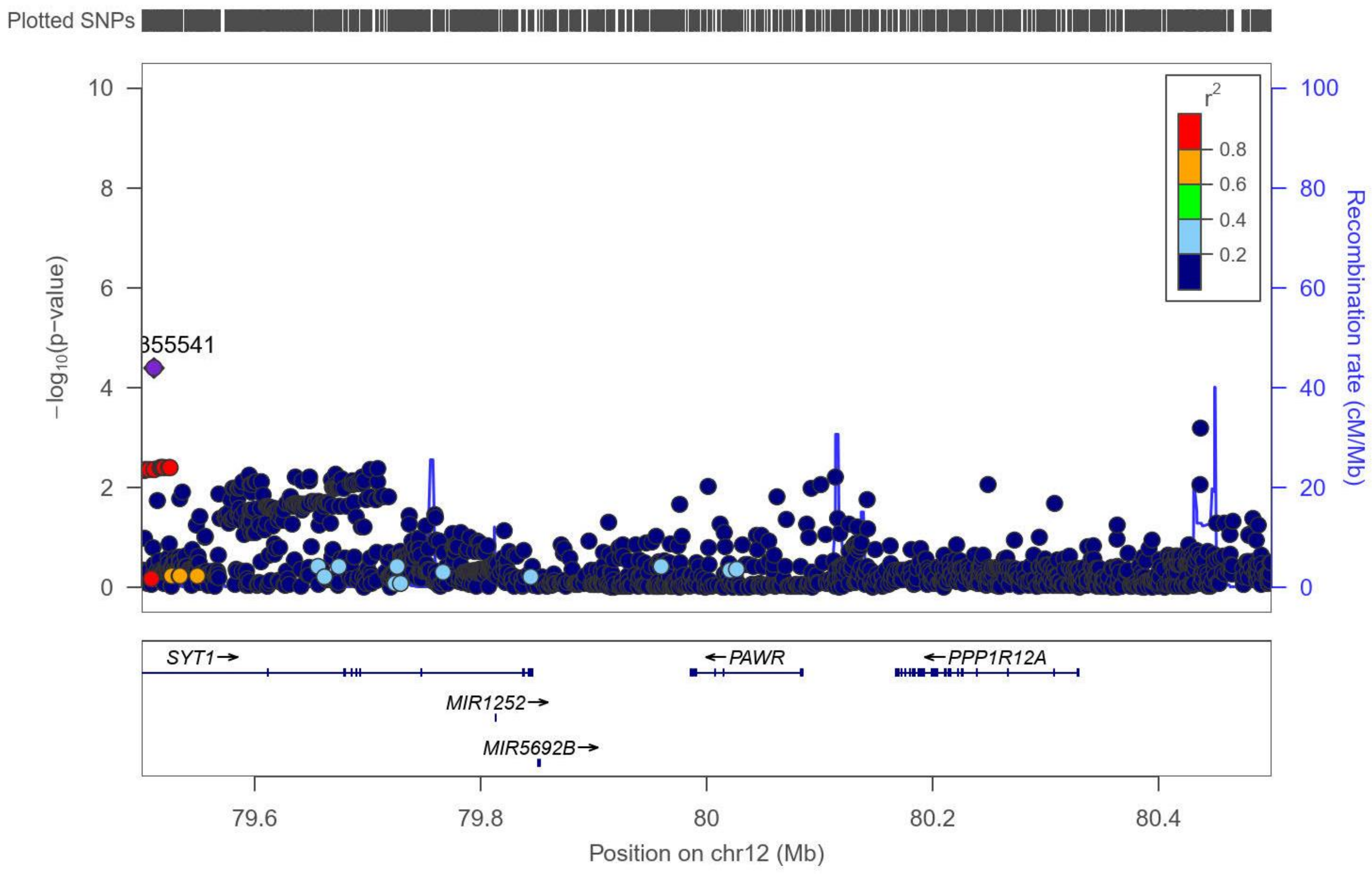

Every dot is a SNP in a 1 Megabase (Mb) window. *X*-axes represent chromosome position of the SNP and the relative genes position in the locus. *Y*-axes represent –$\log_{10}$(*P*-value) of each SNP. Blue peaks indicate the Recombination rate (cM/Mb). The most significant SNP is labelled in purple.

Supplementary Fig. 26: Regional association plots for conditional logistic regression in genome-wide significant locus in chromosome 17.

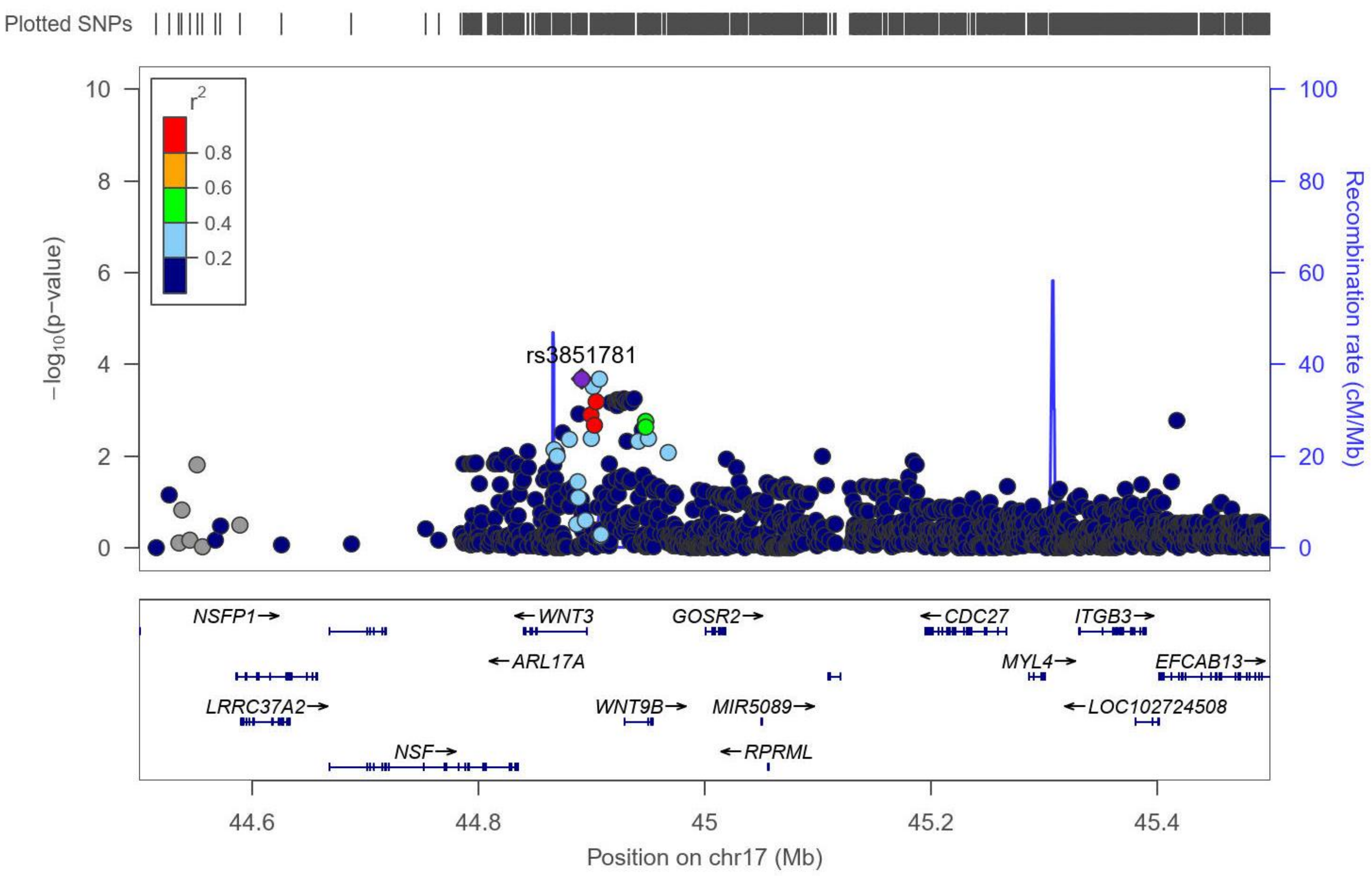

Every dot is a SNP in a 1 Megabase (Mb) window. *X*-axes represent chromosome position of the SNP and the relative genes position in the locus. *Y*-axes represent –$\log_{10}$(*P*-value) of each SNP. Blue peaks indicate the Recombination rate (cM/Mb). The most significant SNP is labelled in purple.

Supplementary Fig. 27: Regional association plots for conditional logistic regression in genome-wide significant locus in chromosome 20.

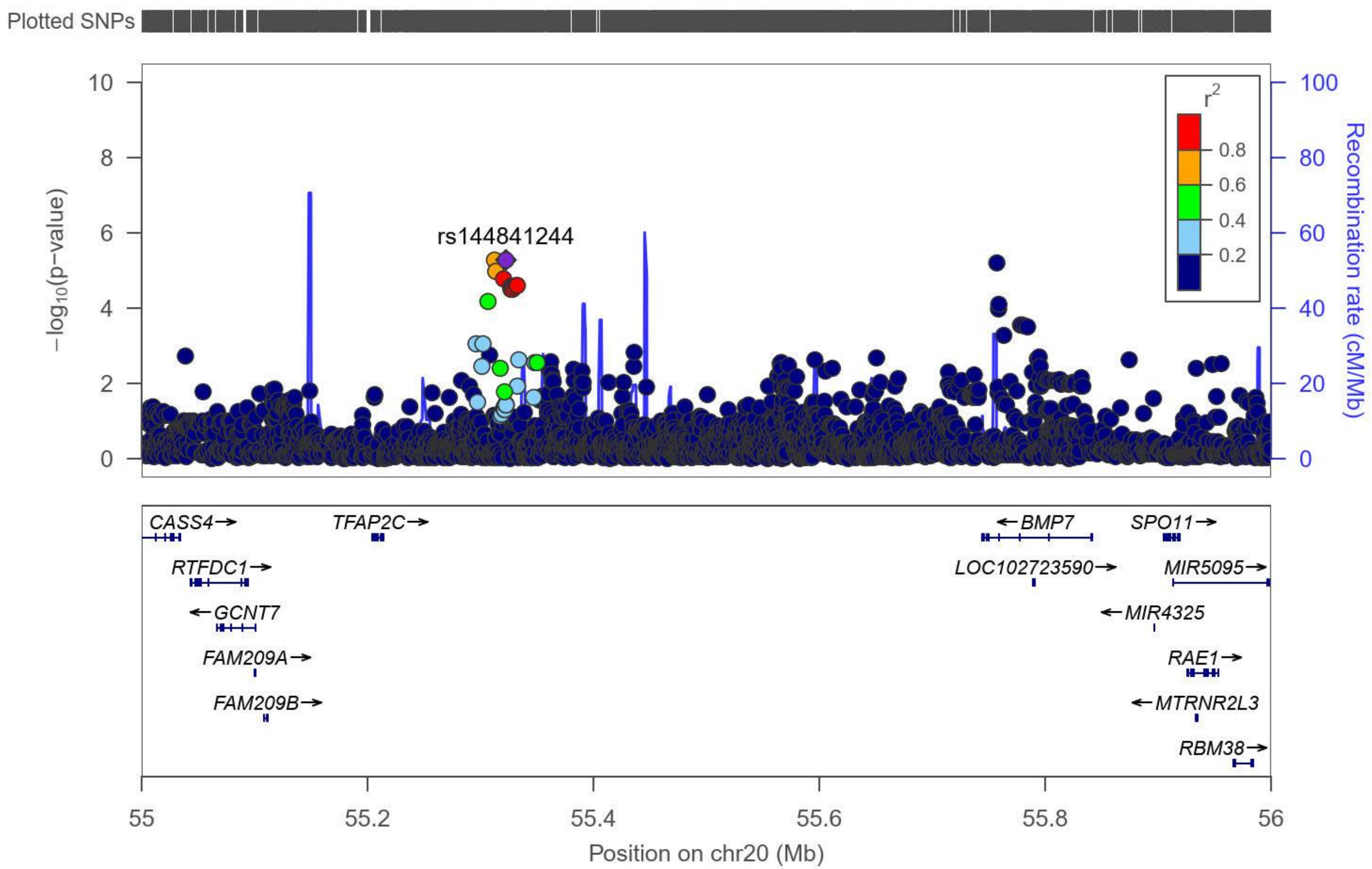

Every dot is a SNP in a 1 Megabase (Mb) window. *X*-axes represent chromosome position of the SNP and the relative genes position in the locus. *Y*-axes represent $-\log_{10}$(*P*-value) of each SNP. Blue peaks indicate the Recombination rate (cM/Mb). The most significant SNP is labelled in purple.

Supplementary Fig. 28. Network analysis of putative candidate genes.

Full STRING network
(edges indicate both functional and physical protein associations)

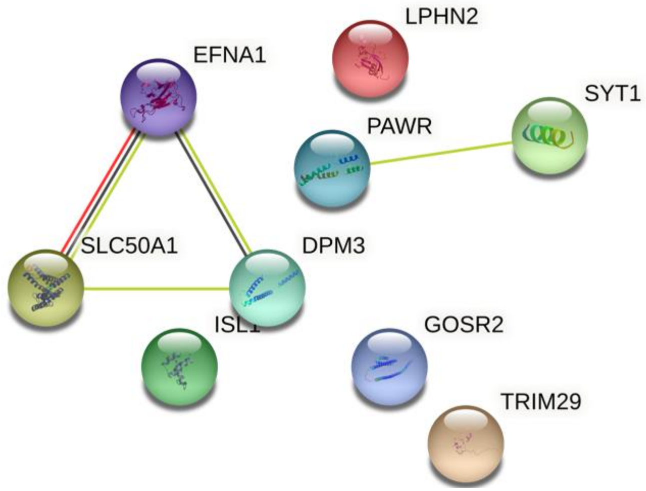

Protein-protein interactions for three proteins/genes other than text mining: **(i)** gene fusions between *EFNA1* and *SLC50A1*, **(ii)** co-expression between *EFNA1* and *DPM3*, **(iii)** and *EFNA1* and *SLC50A1*.

k- means clustering
(network is clustered to a specified number of clusters)

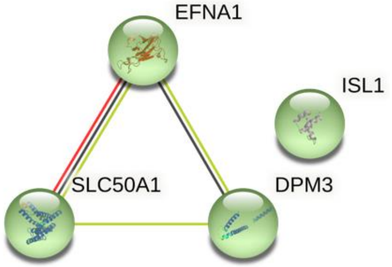

MCL clustering
(network is clustered to a specified "MCL inflation parameter")

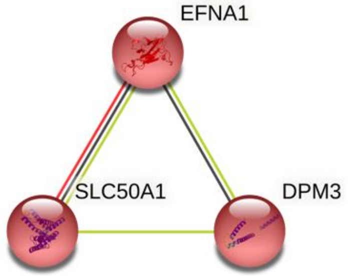

Reference publication (PMID: 29659830): Warrington et al. identified the *EFNA1*-locus, with *EFNA1 DPM3*, *EFNA3*, *KRTCAP2*, and *SLC50A*1 as a locus that could implicate cholesterol metabolism in steroidogenesis as a link to testosterone exerting a role on 2D:4D ratio variation [5].

Number of nodes: 9; number of edges: 4; average node degree: 0.889; avg. local clustering coefficient: 0.556; expected number of edges: 0; PPI enrichment **p-value: 0.000205**

Number of nodes: 4; number of edges: 3 (in both clusters); average node degree: 1.5; avg. local clustering coefficient: 0.75; expected number of edges: 0; PPI enrichment **p-value: 7.38e-0** (based on the included number of genes/proteins = 4)

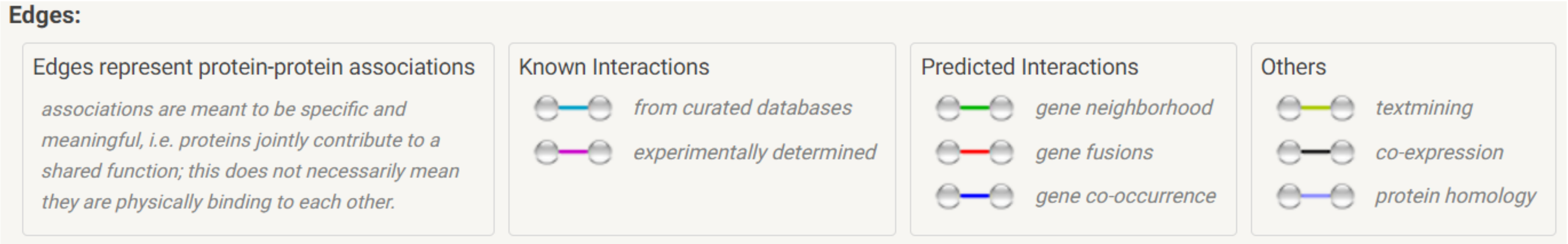

Supplement: Supplementary file 1 — Supplementary Information [file 42003_2022_4092_MOESM1_ESM.pdf]
