# Supplementary material for: A genome-wide association study with tissue transcriptomics identifies genetic drivers for classic bladder exstrophy
Source: Commun Biol. 2022 Nov 9;5:1203. doi: 10.1038/s42003-022-04092-3 (PMC9646906; doi:10.1038/s42003-022-04092-3)
Supplement: Supplementary file 2 — Description of Additional Supplementary Data [file 42003_2022_4092_MOESM2_ESM.docx]

**Description of Additional Supplementary Files**

**File name:** Supplementary Data 1

**Description:** Demography and ethnicity of cancer cell lines used for RNA-seq

**File name:** Supplementary Data 2

**Description:** : All the significant SNP of the Mata Analysis for Classic Bladder Exstrophy. The excel file contains two tables named‚ RR‘ (with relative risks in each sample and in the meta-analysis) and‚ Info_and_FreqRA‘ (with information score and mean dosage of the risk allele in each sample) for all genomewide significant SNPs.
