# Supplementary material for: A genome-wide association study with tissue transcriptomics identifies genetic drivers for classic bladder exstrophy
Source: Commun Biol. 2022 Nov 9;5:1203. doi: 10.1038/s42003-022-04092-3 (PMC9646906; doi:10.1038/s42003-022-04092-3)
Supplement: Supplementary file 5 — Reporting Summary [file 42003_2022_4092_MOESM5_ESM.pdf]

nature portfolio

Corresponding author(s): Enrico Mingardo

Last updated by author(s): Jun 29, 2022

# Reporting Summary

Nature Portfolio wishes to improve the reproducibility of the work that we publish. This form provides structure for consistency and transparency in reporting. For further information on Nature Portfolio policies, see our Editorial Policies and the Editorial Policy Checklist.

## Statistics

For all statistical analyses, confirm that the following items are present in the figure legend, table legend, main text, or Methods section.

| n/a | Confirmed | |
|---|---|---|
| ☐ | ☒ | The exact sample size (*n*) for each experimental group/condition, given as a discrete number and unit of measurement |
| ☐ | ☒ | A statement on whether measurements were taken from distinct samples or whether the same sample was measured repeatedly |
| ☒ | ☐ | The statistical test(s) used AND whether they are one- or two-sided<br>*Only common tests should be described solely by name; describe more complex techniques in the Methods section.* |
| ☒ | ☐ | A description of all covariates tested |
| ☒ | ☐ | A description of any assumptions or corrections, such as tests of normality and adjustment for multiple comparisons |
| ☒ | ☐ | A full description of the statistical parameters including central tendency (e.g. means) or other basic estimates (e.g. regression coefficient) AND variation (e.g. standard deviation) or associated estimates of uncertainty (e.g. confidence intervals) |
| ☒ | ☐ | For null hypothesis testing, the test statistic (e.g. *F*, *t*, *r*) with confidence intervals, effect sizes, degrees of freedom and *P* value noted<br>*Give P values as exact values whenever suitable.* |
| ☒ | ☐ | For Bayesian analysis, information on the choice of priors and Markov chain Monte Carlo settings |
| ☒ | ☐ | For hierarchical and complex designs, identification of the appropriate level for tests and full reporting of outcomes |
| ☒ | ☐ | Estimates of effect sizes (e.g. Cohen's *d*, Pearson's *r*), indicating how they were calculated |

*Our web collection on statistics for biologists contains articles on many of the points above.*

## Software and code

Policy information about availability of computer code

| | |
|---|---|
| Data collection | Microsoft Excel 2021, Graph Pad Pirsm 9, R 4.1.2 |
| Data analysis | Microsoft Excel 2021, Graph Pad Pirsm 9, R 4.1.2 |

For manuscripts utilizing custom algorithms or software that are central to the research but not yet described in published literature, software must be made available to editors and reviewers. We strongly encourage code deposition in a community repository (e.g. GitHub). See the Nature Portfolio guidelines for submitting code & software for further information.

## Data

Policy information about availability of data

All manuscripts must include a data availability statement. This statement should provide the following information, where applicable:

- Accession codes, unique identifiers, or web links for publicly available datasets
- A description of any restrictions on data availability
- For clinical datasets or third party data, please ensure that the statement adheres to our policy

GWAS generated and analysed data during this study are included in this article and its supplementary information files.
The raw RNA-sequencing data of mouse embryonic urinary bladder are deposited at GEO (https://www.ncbi.nlm.nih.gov/geo/query/acc.cgi?acc=GSE190641). The following and temporary secure token has been created to allow the access for reviewing: wfwrwqiubbudpcd
The raw RNA-sequencing data of human embryonic and fetal urinary bladder and genital tissue are deposited at EMBL-EBI expression atlas (https://www.ebi.ac.uk/

arrayexpress/experiments/E-MTAB-6592/).
The raw RNA-sequencing data of cancer cell lines are obtained from EMBL-EBI expression atlas (https://www.ebi.ac.uk/arrayexpress/experiments/E-MTAB-2770/), while raw polyA RNA-seqencing of mature urinary bladder are obtained from GEO (https://www.ncbi.nlm.nih.gov/geo/query/acc.cgi?acc=GSM1067793).
The dataset of RNA-polyA-seq of 38 Muscolar urothelial carcinoma used in this study are available from the corresponding author on reasonable request.

## Human research participants

Policy information about studies involving human research participants and Sex and Gender in Research.

| | |
|---|---|
| Reporting on sex and gender | Only sex has been considered for this study. Gender was not taken in consideration. See supplementary Table 2 for demography and sex in cancer cell lines. |
| Population characteristics | See Supplementary table 3. |
| Recruitment | Patients were recruited upon self consense. |
| Ethics oversight | University of Bonn (Lfd.Nr.031/19). |

Note that full information on the approval of the study protocol must also be provided in the manuscript.

# Field-specific reporting

Please select the one below that is the best fit for your research. If you are not sure, read the appropriate sections before making your selection.

☒ Life sciences ☐ Behavioural & social sciences ☐ Ecological, evolutionary & environmental sciences

For a reference copy of the document with all sections, see nature.com/documents/nr-reporting-summary-flat.pdf

# Life sciences study design

All studies must disclose on these points even when the disclosure is negative.

| | |
|---|---|
| Sample size | Sample size of human embryonic and fetal urinary bladder and genital tissue was limited due to donors availablility. |
| Data exclusions | No data were excluded in this study. For GWAS samples, see Supplementary Information: Principal component analysis. |
| Replication | It is not possible to replicate data of RNA seq of: human embryonic and fetal urinary bladder and genital tissue, mouse embryonic urinary bladder and muscolar urothelial carcinoma tissue due to all of the tissue was used for RNA extraction. Data are depositited in Expression Atlas or GEO (see above, availability statement). |
| Randomization | Partecimpants were allocated in ethnicity groups (see manuscript, supplemtary information, supplementary table 3). |
| Blinding | Blinding is not possible for GWAS since partecipants are divided into control, cases and the respective ethnicity. |

# Reporting for specific materials, systems and methods

We require information from authors about some types of materials, experimental systems and methods used in many studies. Here, indicate whether each material, system or method listed is relevant to your study. If you are not sure if a list item applies to your research, read the appropriate section before selecting a response.

## Materials & experimental systems

| n/a | Involved in the study | |
|---|---|---|
| ☒ | ☐ | Antibodies |
| ☐ | ☒ | Eukaryotic cell lines |
| ☒ | ☐ | Palaeontology and archaeology |
| ☐ | ☒ | Animals and other organisms |
| ☒ | ☐ | Clinical data |
| ☒ | ☐ | Dual use research of concern |

## Methods

| n/a | Involved in the study | |
|---|---|---|
| ☒ | ☐ | ChIP-seq |
| ☒ | ☐ | Flow cytometry |
| ☒ | ☐ | MRI-based neuroimaging |

# Eukaryotic cell lines

Policy information about cell lines and Sex and Gender in Research

| | |
|---|---|
| Cell line source(s) | Data of cancer cell lines are obtained from expression atlas (accession: E-MTAB-6592). Cell line soruce is described in expression atlas E-MTAB-6592. |
| Authentication | Authentication of cell lines is described in E-MTAB-6592 |
| Mycoplasma contamination | Cell lines are not tested for micoplasma contamination |
| Commonly misidentified lines (See ICLAC register) | *Name any commonly misidentified cell lines used in the study and provide a rationale for their use.* |

# Animals and other research organisms

Policy information about studies involving animals; ARRIVE guidelines recommended for reporting animal research, and Sex and Gender in Research

| | |
|---|---|
| Laboratory animals | Mus Muscuslus, SWISS strain. E10.5, E12.5 and E15.5 |
| Wild animals | No wild animals were used in this study. |
| Reporting on sex | No sex was reported for Mous Musculus at E10.5, E12.5, E15.5. |
| Field-collected samples | No Field-collected samples are used in this study. |
| Ethics oversight | Experimental protocols were approved by the institutional committee of the University of Bonn (Lfd.Nr.031/19); Regierungspräsidium Darmstad. |

Note that full information on the approval of the study protocol must also be provided in the manuscript.
